# Genomic Analysis of the Halotolerant Hydrocarbon-Oxidizing Bacterium *Ectopseudomonas guguanensis* G3 from a Petroleum Reservoir

**DOI:** 10.3390/biology15120937

**Published:** 2026-06-16

**Authors:** Alexey P. Ershov, Tatyana P. Tourova, Diyana S. Sokolova, Ekaterina M. Semenova, Tamara N. Nazina

**Affiliations:** Winogradsky Institute of Microbiology, Research Center of Biotechnology, Russian Academy of Sciences, 119071 Moscow, Russia; tptour@rambler.ru (T.P.T.); sokolovadiyana@gmail.com (D.S.S.); semenova_inmi@mail.ru (E.M.S.); nazina@inmi.ru (T.N.N.)

**Keywords:** genomic analysis, oil degradation, hydrocarbon-oxidizing bacteria, halotolerance, *Ectopseudomonas guguanensis*

## Abstract

An inevitable decrease in oil production from reservoirs all over the world necessitates the application of microbial enhancement of oil recovery (MEOR) technologies. The high total salinity of formation water is a factor strongly suppressing the growth of most industry-applicable strains of hydrocarbon-oxidizing bacteria. The halotolerant strain *Ectopseudomonas guguanensis* G3 isolated from an oil reservoir (Republic of Kazakhstan) has demonstrated high efficiency of oil degradation and presumable biosurfactant production. The ability of the strain to utilize crude oil, *n*-alkanes, toluene, and xylene and its resistance to NaCl concentrations up to 6% were shown, as well as a high decrease in the interfacial tension of the culture liquid. Genomic analysis of the strain confirmed its ability to oxidize aromatic oil compounds and a wide range of *n*-alkanes (with a chain length up to C_30_) and revealed its potential capabilities to produce alginate, consume nitrate and urea as nitrogen sources, and synthesize betaine as an osmoprotectant. These findings demonstrate the high potential of *E. guguanensis* strain G3 to be used in oil reservoirs with high-salinity formation water in the biotechnology of oil displacement through oil degradation and in situ microbial metabolite production.

## Simple Summary

Microbial enhancement of oil recovery in reservoirs with high-salinity formation water is limited because application of industrial hydrocarbon-oxidizing strains is often inefficient. Hence, halotolerant bacteria that can inhabit such environments are of great biotechnological potential. *Ectopseudomonas guguanensis* strain G3 has been isolated from an oil reservoir in Kazakhstan and demonstrated its adaptation to osmotic stress and efficient hydrocarbon degradation. Changes in rheological characteristics of the strain G3 culture liquid indicated a probable biosurfactant production process which is a basis of additional oil recovery in the microbial biotechnology. Genetic determinants of these promising physiological traits were studied via whole-genome sequencing. The G3 genome contains genes for halotolerance due to osmoprotectant synthesis and for utilization of aliphatic (*n*-alkanes) and aromatic (toluene, xylene, etc.) oil components. Adaptability of the strain is supported by its broad capabilities for nitrogen compound metabolism, and its biotechnological potential is of interest because of the alginate production pathway’s presence in the genome. Thus, strain G3 is considered to be a halotolerant hydrocarbon-oxidizing bacterium, resistant to harsh environmental conditions due to a highly adaptable metabolism and applicable for microbially enhanced oil recovery in reservoirs with high-salinity formation water because of its presumable oil-displacement metabolite production.

## 1. Introduction

The high total salinity of formation water is a factor significantly limiting bacterial growth in oil reservoirs due to the denaturation of enzymes, degradation of cell walls, and low oxygen solubility [1]. Environmental stress in such extreme habitats combines osmotic stress with the lack of utilizable organic compounds and electron acceptors. Microorganisms able to utilize the components of crude oil under such conditions possess specialized genetic mechanisms such as the production of alkane hydroxylase and/or aromatic oxidation enzymes [2,3,4]. To survive at high salinity, they synthesize osmoprotective compounds, including betaine, ectoine, and hydroxyectoine [5]. Many of these prokaryotes are shown to be polyextremophiles resistant not only to high salinity, but also to high or low temperatures [6,7] and to heavy metals [8,9].

Aerobic halotolerant hydrocarbon-oxidizing bacteria from oil reservoirs with high-salinity formation water mostly belong to the phyla *Pseudomonadota*, *Actinomycetota*, and *Bacillota*. The first group predominantly consists of *Gammaproteobacteria* of the order *Oceanospirillales* and includes the genera *Halomonas* and *Modicisalibacter* from the family *Halomonadaceae* [10,11,12,13], *Oleispira* and *Thalassolituus* from the family *Oceanospirillaceae* [14,15], *Alcanivorax* from *Alcanivoracaceae* [14,16], etc. Other *Gammaproteobacteria* such as *Marinobacter* (order *Alteromonadales*) and *Pseudomonas* (order *Pseudomonadales*) are also widespread in oil reservoirs with high-salinity formation water [7,15,17,18].

Bacteria of the genera *Arthrobacter* and *Amycolicicoccus* (reclassified as *Hoyosella*) of the phylum *Actinomycetota* are known as widespread components of halotolerant microbial communities [4,19,20]. Bacteria of the genera *Bacillus*, *Halobacillus*, and *Halanaerobium* (phylum *Bacillota*) also commonly inhabit oil reservoirs with high-salinity formation water [9,12,21]. Archaea of the genera *Halobacterium*, *Halococcus*, *Haloferax*, and *Haloarcula* of the order *Halobacteriales* (phylum *Methanobacteriota*) have been shown to grow in extremely saline formation water [1,8,22]. Living cells of the listed archaea are able to survive environmental salinity up to 32% NaCl (*w*/*v*), while most halophilic hydrocarbon-oxidizing bacteria cannot grow at a total salinity higher than 20–25% (*w*/*v*) [10,17,23].

Hydrocarbon-oxidizing bacteria possess several molecular mechanisms of oil component utilization. Bacterial alkane hydroxylase genes are well known and described [2,3,4,5,24,25]: alkane 1-monooxygenase AlkB oxidizing alkanes not longer than hexadecane, flavin-dependent monooxygenases AlmA and LadA for long-chain alkanes, and cytoplasmic cytochrome P450 monooxygenase-oxidizing medium-chain alkanes from C_6_ to C_11_. Halotolerant microorganisms are able to utilize many other hydrocarbons as well: *iso*-alkanes [7,17], naphthalene and phenanthrene [19,22], tetracosane [16], crude oil itself [9,15], etc. Total petroleum hydrocarbons (TPHs) were demonstrated to be decreased to 74% at 2.5% NaCl by an *Alcanivorax* strain, to 65% by a *Halobacillus* strain, and to 53–87% by archaeal strains at 6–24% NaCl [9,16,22]. Some of the genes for aromatic oil component degradation are localized in bacterial plasmids, which makes possible the horizontal gene transfer in microbial communities of formation water and distribution of these biochemical pathways among oil-associated prokaryotes of the reservoir [19]. Recent studies where the growth of *Pseudomonas* and *Bacillus* strains modulated the composition of the microbial community toward more effective oil consumption partly confirm this hypothesis [21,26].

Halotolerant hydrocarbon-oxidizing microorganisms are of great practical interest for microbially enhanced oil recovery (MEOR) in oil reservoirs with high-salinity formation water and for bioremediation of oil-polluted ecosystems [27,28,29]. Hydrocarbon-oxidizing bacteria, similar to some other aerobic organotrophic bacteria, produce biosurfactants during their growth on oil and, therefore, reduce surface and interfacial tension between oil and formation water, leading to incremental oil recovery [30,31,32]. Producers of biosurfactants mostly belong to the bacterial phyla *Pseudomonadota* [5,11], *Actinomycetota* [33,34], and *Bacillota* [6,21]. Field trials of MEOR technologies are performed regularly [34,35,36,37], which confirms the fundamental and applied significance of investigating aerobic organotrophic bacteria isolated from oil reservoirs with high-salinity formation water.

This work is a part of the investigation of the oil reservoir (Republic of Kazakhstan) with high-salinity formation water in order to determine the potential for MEOR application. The aim of the current study was to examine the degradation of oil under high-salinity conditions by the hydrocarbon-oxidizing bacterial G3 strain isolated from the oil reservoir and to investigate the genomic determinants of this process.

## 2. Materials and Methods

### 2.1. Sampling and Isolation of the Bacterial Strain

Strain G3 was isolated from a formation water sample collected in 2021 in the oil reservoir (Republic of Kazakhstan) located at a depth of 1920–2010 m below sea level. The oil of the reservoir was characterized by a density of 0.74–0.77 g·cm^−3^ (at 20 °C) and viscosity of 1.75–2.74 mPa·s and possessed a high content of solid hydrocarbons: paraffins—13.7%, resins—8.7%, and asphaltenes—1.0%. Formation water of the calcium chloride type had a total salinity of 64–80 g·L^−1^. To maintain formation pressure at the oil field, coproduced water separated from oil was used for injection.

Aerobic enrichment cultures were obtained by inoculating produced water into liquid TEG medium containing bacto-tryptone (5.0 g), yeast extract (2.5 g), glucose (1.0 g), NaCl (5.0 g), and distilled water (1 L); pH 7.0–7.2. The serial dilution technique was used for the strain isolation. Bacteriological agar (20 g·L^−1^) was added to the medium in order to obtain single colonies that were subsequently re-inoculated into the liquid medium. The 16S rRNA gene sequence analysis was performed to confirm the purity of the isolated strain. The hydrocarbon degradation activity of the strain was verified in a liquid medium with oil. The strain G3 has been deposited at the Collection of Unique and Extremophilic Microorganisms (UNIQEM; Moscow, Russia) under the number UQM 42133.

### 2.2. Phenotypic Characterization

Physiological and morphological characteristics of the strain were studied in the TEG medium. Different temperatures (5, 10, 15, 22, 30, 37, 42, 47, and 55 °C), salt concentrations (0, 2, 5, 15, 30, 60, 90, 120, and 150 g·L^−1^ NaCl), and pH (5.0–10.5 with increments of ~0.5 pH units) were used to determine the optimal conditions for the growth of the strain in the TEG medium for 7 days.

Mineral medium containing K_2_HPO_4_ (1.5 g), NH_4_Cl (1.0 g), KH_2_PO_4_ (0.75 g), MgSO_4_·7H_2_O (0.2 g), KCl (0.1 g), CaCl_2_·2H_2_O (0.02 g), NaCl (30 g), pre-made trace element solution (10 mL) [38], and distilled water (1 L), pH 7.0–7.2, was used to study the utilization of oil alkanes and rheological characteristics of culture liquid with different organic substrates. Carbon and energy sources were added to the medium from sterilized stock solutions in the following final concentrations: glucose—5 g/L; sodium fumarate—2 g/L; ethanol—2 mL/L; and glycerol—2 mL/L. Sterilized heavy crude oil with a density of 0.932 kg/m^3^ (20 °C, in surface condition) was added at a concentration of 1000 µL·L^−1^ [39].

The morphology of the cells was examined under a scanning electron microscope (Quattro S, Thermo Fisher Scientific Brno s.r.o., Brno-Černovice, Czech Republic) under high vacuum after biomass preparation as described previously [40].

### 2.3. Analytical Methods

An Ultrospec 2100 pro spectrophotometer (Amersham Biosciences, Amersham, Buckinghamshire, UK) was used to estimate the changes in biomass’s optical density at 600 nm. The pH of the cultivation media was measured with a Seven Compact S220 pH meter (Mettler Toledo, Greifensee, Switzerland). Rheological characteristics of the media, i.e., surface tension (between the medium and air) and interfacial tension (medium/*n*-hexadecane), were measured with a Surface Tensiomat 21 semi-automatic tensiometer (Cole-Parmer, Vernon Hills, IL, USA) by the ring separation method at 25 °C.

The utilization of oil alkanes was measured chromatographically. Extraction of the non-polar compounds of the culture media was performed with *n*-hexane, and silica gel columns were used for their fractioning. The profiles of light aliphatic hydrocarbons were determined using a Crystal 5000.1 gas chromatograph (Chromatec, Yoshkar-Ola, Mari El, Russia) with a ZB-FFAP 15 m capillary column and a flame ionization detector. All measurements were performed in triplicates; sterile medium with the same amount of crude oil was used as an abiotic control. Phytane (iC20) was used as an internal standard due to its high resistance to biodegradation [41]. Utilization of oil alkanes was estimated using the internal normalization method based on the total *n*-alkanes-to-isoprenoids ratio.

Analysis of total petroleum hydrocarbons (TPH) in oil samples was performed in triplicates using an AN-2 oil product content analyzer (Neftekhimavtomatika, Saint Petersburg, Russia) by the extraction-photometric method [42]. Extraction of non-polar compounds from 100 mL of the culture liquid was carried out using 20 mL of perchloroethylene (PCE), and then 0.2 mL of the non-polar fraction was diluted with 1.8 mL of PCE. The content of oil products in the samples was determined photometrically in the infrared region of the spectrum according to the manufacturer’s recommendations.

### 2.4. DNA Extraction and 16S rRNA Gene Sequencing

The DNA extraction from the strain’s biomass was performed using the Diatom™ DNA Prep 100 kit (Isogen Laboratory, Moscow, Russia) with the standard manufacturer’s protocol. For the PCR, the purified DNA was used as the template and Bacteria-universal primer set 27F–1492R of 16S rRNA gene was used for the strain identification [43]. DNA sequencing was performed according to the Sanger method on an ABI 3730 DNA Analyzer automatic sequencer using the ABI PRISM^®^ BigDye™ Terminator v.3.1 reagent kit (Applied Biosystems, Waltham, MA, USA). Taxonomic assignment of the strain was performed with the EzBioCloud online service [44].

### 2.5. Genome Sequencing and Analysis

The QIAamp DNA Mini Kit (QIAGEN, Germantown, MD, USA) was used for extraction of the genomic DNA of the strain. Construction of the DNA libraries was performed with the NEBNext DNA library prep reagent set for Illumina with the standard protocol. Sequencing of genomic DNA was carried out using the HiSeq 2500 platform (Illumina, Inc., San Diego, CA, USA) with 150 bp paired-end reads. The following software was applied for the downstream analysis: FastQC v.0.12.0 (http://www.bioinformatics.babraham.ac.uk/projects/fastqc/ (accessed on 22 January 2025)) for the raw reads’ quality check; Trimmomatic v.0.39 [45] with the default settings for paired-end reads for low-quality reads’ trimming; SPAdes v.3.13.0 [46] with the default settings for the de novo assembly of the quality-filtered reads; QUAST v.5.0 [47] for the resulting assembly’s quality assessment; QualiMap 2 v.2.2.2 [48] and Bowtie 2 v.2.3.5.1 [49] for the genome coverages’ estimation; NCBI Prokaryotic Genome Annotation Pipeline (PGAP) v.4.7 [50] for the genome annotation.

Phylogenetically closely related whole-genome sequences from GenBank were analyzed to confirm the assignment of the strain at the species level as recommended by Chun et al. [51]. Average nucleotide identity (ANI) values were determined using FastANI v.1.3 [52]. A whole-genome-based phylogenetic tree was generated using the CodonTree method within BV-BRC v.3.54.6a [53], which used PGFams as homology groups. A total of 500 PGFams were found among selected genomes using CodonTree analysis, and the aligned proteins and coding DNA from single-copy genes were used for RAxML analysis [54]. iTOL v.7.2.2 [55] was used for tree visualization.

Pangenome analysis was performed using the bioinformatic pipeline IPGA v.1.09 [56]. The reconstruction of possible metabolic pathways was carried out based on comparison of the genome of strain G3 using the BlastKOALA (v.3.1) tool of KEGG [57], MetaCyc v.29.1 [58], and BV-BRC. The circular genome map of strain G3 was constructed using the Proksee web service release v1.0.0a6 [59]. To predict the possible events of horizontal gene transfer (HGT), the online service IslandViewer release 4 was used [60]. The neighborhood estimation and visualization of benzoate, catechol, and xylene degradation genes were performed using CAGECAT release 1.0 [61]. Gene clusters were drawn using Proksee and the online service Gene Graphics v.2.02 [62].

### 2.6. Nucleotide Sequence Accession Numbers

The GenBank/EMBL/DDBJ accession numbers for the 16S rRNA gene sequence and the genomic assembly of strain G3 are PX970282.1 and GCF_047468755.1 (JBLGTS000000000.1), respectively.

## 3. Results and Discussion

### 3.1. Isolation, Identification, and Physiological Investigation of Strain G3

The strain G3 was isolated from formation water of an oil reservoir in Kazakhstan in the course of screening experiments. The strain grew in a mineral medium with heavy oil as the sole source of carbon and energy, significantly decreased surface and interfacial tension of the culture liquid during growth with several tested organic substrates (Table 1). All measurements were performed in triplicates. Robustness of the changes in rheological characteristics was estimated with one-way ANOVA (*F* (5, 12) = 61.1, *p* < 0.001 for surface tension; *F* (5, 12) = 110.0, *p* < 0.001 for interfacial tension). Post hoc Tukey’s HSD test confirmed that all experimental variants significantly differed from abiotic control (*p* < 0.001 for all pairwise comparisons). Therefore, the strain G3 was chosen for this study and, on the basis of 16S rRNA gene sequence analysis, was identified as a strain of *Ectopseudomonas guguanensis* with 100.0% identity to the gene of the *E. guguanensis* type strain CC-G9A^T^.

**Figure 1:**
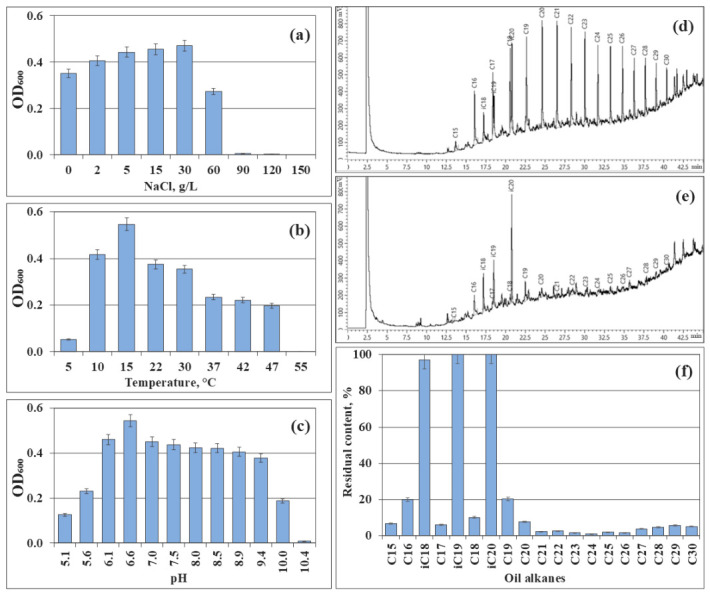
Growth profiles of *E. guguanensis* strain G3 in the TEG medium at various NaCl concentrations (**a**), temperatures (**b**), and pH (**c**); chromatograms of the saturated hydrocarbon fraction of sterile oil (**d**) and oil degraded by strain G3 (**e**), and residual content of alkanes (%) in oil degraded by strain G3 (**f**) after incubation in a liquid medium with crude oil at 30 °C for 30 days. Designations: iC18, iC19, and iC20, *iso*-alkanes.

Cells of the strain were rods, 1.0–1.9 × 0.3–0.4 μm (Figure S1). Phenotypic characteristics of the strain, including ranges of NaCl concentrations, temperature, and pH for its growth, are shown in Figure 1a–c. G3 grew at 5–47 °C (optimum, 15 °C), at pH 5.1–10.0 (optimum, 6.6), and at NaCl concentrations of 0–60 g·L^−1^ (optimum, 30 g·L^−1^). Growth in media supplemented with more than 60 g NaCl·L^−1^ was weak, but the total salinity of the formation water sample (64–80 g·L^−1^) indicates that G3 is able to survive at higher salt concentrations. The strain could use carbohydrates (glucose, fructose), organic acids (acetate, pyruvate, fumarate), alcohols (methanol, ethanol, glycerol), oligopeptides (peptone, tryptone), molasses, aromatic compounds (toluene, xylene), and *n*-alkanes as carbon and energy sources.

The genus *Ectopseudomonas* was described in 2024 [18], so there is a critical lack of data on its physiology, but compared to other hydrocarbon-oxidizing strains of the *Pseudomonas* clade, *E. guguanensis* G3 has a remarkably higher optimum of growth with different NaCl concentrations (30 g·L^−1^). In our previous studies, none of eight *Pseudomonas* isolates had a maximal growth rate in media supplemented with more than 20 g NaCl·L^−1^ [41,63]; in a recent study, only 9 of 43 tested *Pseudomonas* strains had positive growth results with 40 g NaCl·L^−1^ [64]. Many hydrocarbon-oxidizing *Pseudomonas* strains have recently been isolated from cold environments, and none of them could grow at temperatures above 37–40 °C [18,65,66]. These comparisons clearly demonstrate the width of the isolate’s growth ranges, indicating its ecological adaptability and potential for its industrial application in different environmental conditions.

*E. guguanensis* G3 demonstrated hydrocarbon-oxidizing ability in a liquid medium with crude oil as a carbon source. Analysis of degraded oil samples showed that the strain effectively consumed C_15_–C_30_ *n*-alkanes, drastically decreasing their total residual content to 4.2% (Figure 1d–f). In a recent study, a *Pseudomonas* strain utilized only 26–84% of different alkane fractions under similar conditions but with only 10 g NaCl·L^−1^ in a mineral medium [67]. The TPH content of heavy oil decreased to 84.7% (two-tailed Student’s *t*-test, *t* (4) = 3.62, *p* = 0.022) during 30 days of cultivation at 30 °C compared to the sterile control sample. The isolate reduced the surface and interfacial tension of its culture liquid by 22.0 ± 1.2 mN·m^−1^ and 37.3 ± 1.1 mN·m^−1^, respectively, which is strong evidence of biosurfactant synthesis by the strain. To investigate the mechanisms underlying these processes, the whole genome of *E. guguanensis* G3 was sequenced and analyzed.

### 3.2. Genomic, Phylogenomic, and Pangenomic Analyses of Strain G3

The genome of the *E. guguanensis* strain G3 consists of 5,356,018 bp and comprises 82 scaffolds with an N_50_ value of 121,736 bp, G+C content of 64.05%, and coverage of 243×. Completeness of the genome is 99.5%, and contamination is equal to 0.5%. Analysis of ANI similarity between the G3 genome and those of phylogenetically closely related strains (Figure S2) showed its highest similarity with the genome of *E. guguanensis* strain SRIHER B649 (GenBank assembly GCF_031460255.1), which confirmed the taxonomic assignment of the strain G3 to this bacterial species. Comparison of these strains (Table S1) showed that only the strain SRIHER B649 was isolated from the hydrocarbon-containing environment (oil-contaminated marine water) enforcing our assumption of the unique adaptability of the strain G3 to the harsh conditions of the oil reservoir and the potential for its application in MEOR.

In the G3 genome, 4965 genes are annotated by NCBI RefSeq; 4831 are protein-coding genes, 69 are classified as pseudogenes, and 65 encode different types of RNA. According to BV-BRC PATRIC analysis, 42% of the genes are distributed into 11 subsystem super classes (Figure S3), the largest of which are metabolism (891 genes, including 283 for “amino acids and derivatives” and 276 for “cofactors, vitamins, prosthetic groups”), energy (302 genes consisting of 156 for “respiration” and 146 for “energy and precursor metabolites generation”), protein processing (243 genes, including 178 for “protein synthesis”), membrane transport (214 genes), and cellular processes (192 genes).

Analysis of the enzymatic composition of metabolic pathways, based on the results of functional protein prediction using the BlastKOALA service, suggests that the G3 genome contains all the key genes responsible for the complete carbohydrate metabolism pathways: glycolysis, pyruvate oxidation, TCA cycle, pentose phosphate pathway (oxidative and non-oxidative phases), Entner–Doudoroff pathway, and methylcitrate cycle. The G3 genome also contains the key enzymes for complete lipid metabolism pathways (fatty acid biosynthesis and beta-oxidation). Furthermore, the genes for complete biosynthesis pathways of a wide range of vitamins and cofactors were annotated in the genome: riboflavin, pyridoxal-P, NAD, coenzyme A, pimeloyl-ACP, biotin, lipoic acid, molybdenum cofactor, PreQ1, siroheme, heme, cobalamin, and ubiquinone. The G3 genome carries the genes *cys*ND, *cys*C, *cys*H, and *cys*I, responsible for assimilatory sulfate reduction.

The “central metabolism” branch of the “energy and precursor metabolites generation” class includes the TCA cycle genes that are typical for aerobic organotrophic bacteria. These genes of the strain G3 encode aconitate hydratase AcnAB (EC: 4.2.1.3), citrate synthase (si) GltA (EC: 2.3.3.1), isocitrate dehydrogenase Icd (EC: 1.1.1.42), malate:quinone oxidoreductase Mqo (EC: 1.1.5.4), succinate dehydrogenase flavoprotein subunit SdhA (EC: 1.3.5.1), succinyl-CoA ligase alpha and beta chains SucCD (EC: 6.2.1.5), etc. The “central metabolism” subsystem also includes the genes of the pentose phosphate and Entner–Doudoroff pathways, such as *edd* for encoding phosphogluconate dehydratase (EC: 4.2.1.12), *eno* for enolase (EC: 4.2.1.11), *gap* for glyceraldehyde-3-phosphate dehydrogenase (EC: 1.2.1.12), *pgl* for 6-phosphogluconolactonase (EC: 3.1.1.31), *pyk* for pyruvate kinase (EC: 2.7.1.40), *rpe* for ribulose-phosphate 3-epimerase (EC: 5.1.3.1), *rpiA* for ribose-5-phosphate isomerase A (EC: 5.3.1.6), *tal* for transaldolase (EC: 2.2.1.2), *tkt* for transketolase (EC: 2.2.1.1), and *zwf* for glucose-6-phosphate 1-dehydrogenase (EC: 1.1.1.49), etc. The presence of these genes in the genome makes the metabolism of strain G3 adaptable to harsh environmental conditions, similar to other *Pseudomonas* bacteria [68,69,70].

To confirm the phylogenetic position of the strain with high robustness, a phylogenetic tree was constructed based on 100 single-copy proteins using the Bacterial Genome Tree Service of the BV-BRC portal (Figure S4). Eight genomes of *E. guguanensis*, including the genome of the studied strain G3, were used for the analysis, as well as genomes of type strains of other species represented in the BV-BRC (PATRIC) database. In the phylogenomic tree, strain G3 was included in a common cluster with other strains of the species *E. guguanensis*. A high ANI value (97.5%) corresponded to the intraspecies criterion [51,71], which confirmed its affiliation to this species.

**Figure 2:**
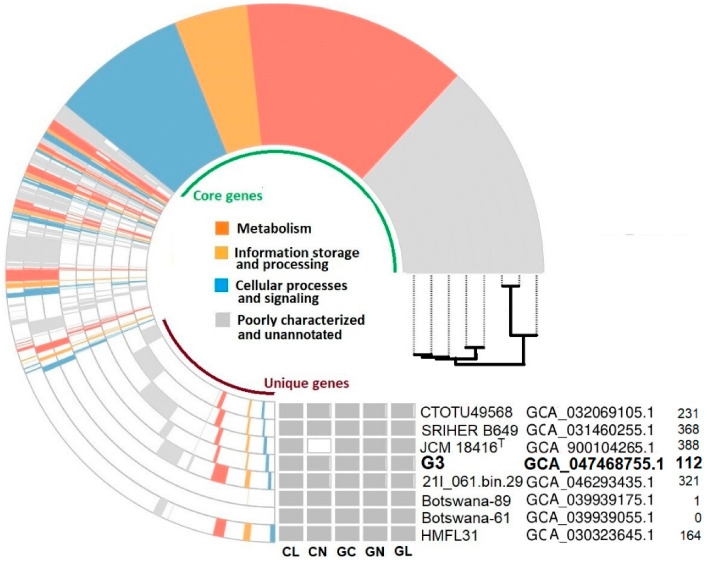
Pangenome analysis of 8 strains of the *E. guguanensis* species calculated with IPGA. Primary COG annotation showing the core genes, unique genes, completeness (CL), contigs number (CN), GC content (GC), gene number (GN), and genome length (GL). The numbers show the quantity of unique genes in each genome.

The genomes of *E. guguanensis* strains, including the G3 genome, were used in a pangenome analysis conducted using the IPGA service. When considering these closely related strains of the same species, the proportion of core genes of all orthologous gene clusters (GCs) in this analysis was equal (55.3%), while the number of unique genes sequenced in different genomes dropped to 2.3% (Figure 2). The number of unique genes in each genome ranged from 1 to 388. The primary COG annotation showed that 112 unique genes in the pangenome result for strain G3 included 19 metabolic genes, 23 cellular process and signaling genes, 11 information storage and processing genes, and 58 unannotated and poorly characterized genes. However, only 23 of these GCs, unique to other *E. guguanensis* strains, had a function predicted by Prokka, KEGG, and BV-BRC (PATRIC) annotations.

This unique gene set underscores the specialized adaptations of strain G3, particularly in aromatic hydrocarbon uptake, biosynthesis of rare sugars, and osmoadaptation. Only in its genome among all analyzed ones, the *xyl*C gene (ACMHYQ_23235) encoding benzaldehyde dehydrogenase (EC: 1.2.1.28) is annotated, which plays a key role in the catabolic pathways for the degradation of such aromatic compounds as xylene and toluene. The strain’s genome also carries the unique *gmd*-*tld* tandem genes (ACMHYQ_02160-02165), which determine the biosynthesis of GDP-6-deoxy-D-talose, an unusual sugar that has been identified as a constituent of only a few microbial polysaccharides. In rare cases, extracellular polysaccharides are found that consist only of 6-deoxy-D-talose [72,73]. The unique gene *pro*P (ACMHYQ_07255) encodes an L-proline/glycine betaine transporter ProP, which promotes osmoadaptation of cells by importing compatible solutes (osmolytes), such as glycine betaine and L-proline, in response to hyperosmotic stress [74].

The circular map of the G3 genome contigs (Figure S5) obtained using the Proksee server shows the localization of single genes and gene operons presumably identified by the analysis carried out in this work. For comparison with the G3 genome, the map shows data from build-in BLAST (v.1.6.1) analysis with the genomes of seven other *E. guguanensis* strains. The results of this comparison show that the G3 genome contains some regions that are not represented in the genomes of other similar strains, with the GC content of these regions different from the average genomic one, which may indicate that they were acquired via horizontal gene transfer.

### 3.3. Genes of Alkane Monooxygenases

The obtained physiological data indicated the ability of strain G3 to grow on *n*-alkanes as the sole sources of energy and carbon. According to the results from the BlastKOALA and BV-BRC portals, the G3 genome contains annotated genes for all enzymes involved in *n*-alkane degradation (Figure S6). In the genome, genes for alkane 1-monooxygenase (EC: 1.14.15.3), *alk*B1 (ACMHYQ_17835) and *alk*B2 (ACMHYQ_19650), with lengths of 1164 bp and 1134 bp, respectively, and the *alm*A (ACMHYQ_22745) gene with a length of 1485 bp, were annotated. Thus, for strain G3, the presence of two forms of the enzyme is assumed: both a non-heme iron-containing form (AlkB) and a flavin-dependent form (AlmA), which act both at the initial stage of *n*-alkane oxidation and at the terminal stage of hydroxylation of fatty acids into omega-hydroxy fatty acids.

In the G3 genome, the genes for alkane-1 monooxygenase are located in different regions of the genome and are not associated with other *n*-alkane degradation genes: one rubredoxin gene *rub*A (ACMHYQ_12545) is located separately, while the other is in tandem with the *rub*B gene (ACMHYQ_15050-15055) of rubredoxin reductase (EC: 1.18.1.1). The genes homologous to the *alk*B2 gene of strain G3 were present in the genomes of all *E. guguanensis* strains and in most genomes of strains of the entire genus *Ectopseudomonas* (*P. oleovorans* clade), while a double set of *alk*B1 genes was detected less frequently.

The multiplicity of *n*-alkane degradation genes has been identified in the genomes of many bacteria of the family *Pseudomonadaceae* and has been most thoroughly studied in *P. aeruginosa* strains [75,76]. The main functional components of the alkane hydroxylase system of *P. aeruginosa* PAO1 included two alkane 1-monooxygenase (*alk*B1 and *alk*B2), two rubredoxin (*rub*A1 and *rub*A2), and one rubredoxin reductase (*rub*B) gene. AlkB1 and AlkB2 alkane 1-monooxygenases have overlapping substrate-length profiles: C_16_ to C_24_ and C_12_ to C_22_ alkanes, respectively. The expression of the *alk*B2 gene is highest in the early exponential phase of growth, and when growth decreases, the *alk*B1 gene is induced.

**Figure 3:**
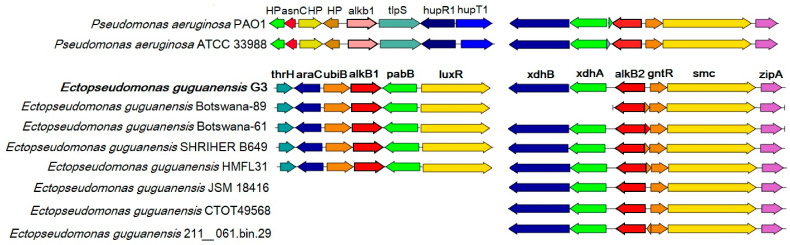
Comparison of *alk*B-regions with flanking genes from the genomes of *E. guguanensis* and *P. aeruginosa* strains. Abbreviations: *alk*B1, alkane 1-monooxygenase AlkB1; *tps*, methyl-accepting chemotaxis sensor/transducer protein; *hup*R1, two-component system response regulator protein; *hup*T1, putative two-component sensor; HP, hypothetical proteins; *par*B, para-aminobenzoate synthase, aminase component; *lux*R, LuxR family transcriptional regulator; *ubi*B, 2-polyprenylphenol hydroxylase; *ara*C, AraC family transcriptional regulator; *alk*B2, alkane 1-monooxygenase AlkB2; *gnt*R, GntR family transcriptional regulator; *smc*, chromosome partition protein SMC; *xdh*A, xanthine dehydrogenase iron–sulfur subunit; *xdh*B, xanthine dehydrogenase, molybdenum binding subunit. Homologous genes are highlighted in one color.

Recently, a similar temporal pattern of expression of alkane 1-monooxygenase genes (*alk*B) was shown in the efficient *n*-alkane degrader *P. aeruginosa* ATCC 33988 isolated from jet fuel [25]. The gene organization of the *alk*B2 regions in the genomes of strain G3 and these *P. aeruginosa* strains was similar, with the *alk*B2 gene sequences having the same length and a high identity level of their translated amino acid sequences (86%), so similar functionality can be assumed for them. The functional characteristics of the *alk*B1 gene product from the G3 genome, which differed significantly from that of *P. aeruginosa* PAO1 and ATCC 33988 (different length and 40–41% identity), remain uncertain (Figure 3).

Comparative characterization of medium- and long-chain *n*-alkane degradation genes was performed for *P. aeruginosa* SJTD-1 [77]. In addition to the *alk*B1 and *alk*B2 genes responsible for the degradation of medium-chain *n*-alkane, the *alm*A gene of flavin-binding monooxygenase was identified in the genome of this strain, the expression of which was induced by long-chain *n*-alkanes (C_18_–C_24_). Since in our work it was shown that strain G3 effectively decomposes long-chain alkanes (C_18_–C_30_), it can be assumed that the flavin-binding monooxygenase, encoded by the *alm*A gene found in its genome, is also responsible for this process. Thus, the genetic determinants found in the G3 genome elucidate the demonstrated efficiency of *n*-alkane degradation by the isolate.

### 3.4. Genes of Aromatic Compound Metabolism

Benzoate, especially in the form of benzoic acid or its salts, plays an important role in the degradation of aromatic hydrocarbons by microorganisms. Many bacteria, including members of the genus *Pseudomonas*, catabolize benzoate under aerobic conditions via hydroxylation to catechol. The G3 genome contains the annotated benzoate degradation operon *ben*ABCD/*xyl*XYZL (ACMHYQ_23325-23310) encoding benzoate/toluate 1,2-dioxygenase (EC: 1.14.12.10/1.14.12.-) and dihydroxycyclohexadiene carboxylate dehydrogenase (EC: 1.3.1.25/1.3.1.-). The catechol (1,2-dihydroxybenzene) formed as a result of the action of these enzymes is an intermediate product in the catabolism of many aromatic compounds to Krebs cycle intermediates.

In the G3 genome, the *xyl*EGFJQKIH operon (ACMHYQ_23300-23265) is annotated, which determines the degradation of catechol via the *meta*-cleavage pathway, whose key enzyme is catechol 2,3-dioxygenase (EC: 1.13.11.2), encoded by the *xyl*E gene (Figure S7). It should be noted that the *xyl*E gene is used in biosensors for detection of aromatic pollutants, since the product of the reaction it determines (2-hydroxymuconate semialdehyde) has a yellow color [78]. The expression of the *xyl*EGFJQKIH operon is induced by substrates (e.g., benzoate, toluate) through the activation of regulatory proteins encoded by the *xyl*R (ACMHYQ_23255) and *xyl*S (ACMHYQ_23260) genes. The set of genes for the degradation of benzoate and catechol in the G3 genome is unique among the *E. guguanensis* strains. The *ben*ABCD operons were annotated only in the genomes of the CTOTU49568, 21I_061.bin.29 and SRIHER B649 strains; however, for further catabolization of catechol, the genes of the *ortho*-cleavage pathway, not the *meta*-pathway, are annotated in them. Besides the G3 genome, genes for enzymes of the catechol *meta*-cleavage degradation pathway are annotated only in the genome of strain HMFL31; however, it does not contain *ben*-operon genes, and catechol formation in this strain apparently occurs via other pathways.

**Figure 4:**
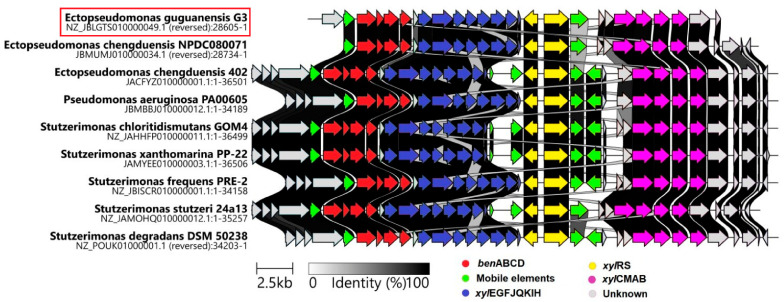
Gene synteny of benzoate, catechol, and xylene degradation in the G3 genome and representative genomes of *Ectopseudomonas*, *P. aeruginosa* and *Stutzerimonas* strains. Genes associated with the annotated genes are highlighted in colors, and unknown genes are shown in gray. Abbreviations: *ben*ABCD, benzoate degradation operon; *xyl*EGFJQKIH, catechol degradation operon; *xyl*RS, regulatory genes; *xyl*CMAB, xylene and toluene degradation operon. The studied strain is highlighted with a red frame.

In *Pseudomonas* spp., the *meta*-cleavage pathway of catechol is often associated with the operons that control the degradation of such aromatic hydrocarbons as toluene and xylene. In the G3 genome, the *xyl*CMAB operon (ACMHYQ_23240-23225) is annotated, encoding benzyl alcohol dehydrogenase (EC: 1.1.1.90), toluene methyl-monooxygenase (EC: 1.14.15.26), and benzaldehyde dehydrogenase (NAD) (EC: 1.2.1.28), which catabolize xylene and toluene to benzoate. Homologous operons were not found in the genomes of other *E. guguanensis* strains, suggesting that the ability to degrade toluene and xylene is unique to the strain G3. According to data from the CAGECAT service, homologous gene structures for aromatic hydrocarbon degradation are mainly annotated in the genomes of *P. aeruginosa* strains and various species of the genus *Stutzerimonas*, and only in some strains of the *E. chengduensis* species (Figure 4).

In the genomes of many bacteria of the genus *Pseudomonas*, the operons of the *meta*-pathway for catechol and xylene/toluene degradation are located on TOL plasmids (e.g., pWW0), which presumably facilitates their horizontal transfer [78]. In the chromosomal region of strain G3, where these operons are located, several mobile elements are present, so it can be assumed that the appearance of these operons only in the genome of strain G3 among the *E. guguanensis* strains occurred as a result of transfer from the genomes of other pseudomonads. This assumption is confirmed by the results of the Alien Hunter module built into the Proksee service and the IslandViewer service, which predict the expected events of horizontal gene transfer (Figure S8).

There is evidence that the ability to degrade xylene and toluene is maintained when glycerol is used as a substrate for growth. In particular, proteomic studies of *P. putida* KT2440 grown on glycerol showed increased expression of proteins associated with the metabolism of aromatic compounds [79]. The possibility of glycerol consumption by strain G3 is confirmed by the presence of the genes *glp*K (ACMHYQ_16780; ACMHYQ_05580) and *glp*D (ACMHYQ_16765; ACMHYQ_05585), which encode the enzymes glycerol kinase (EC: 2.7.1.30) and glycerol-3-phosphate dehydrogenase (EC: 1.1.5.3), and according to MetaCyc, constitute the glycerol degradation pathway I.

The ability of strain G3 to utilize a wide range of aromatic oil compounds confirms its adaptation to the harsh environmental conditions of formation water (Table S1). The diversity of genetic determinants makes *E. guguanensis* G3 a significant component of microbial communities in oil reservoirs as well as other bacteria of the *Pseudomonas* phylogenetic cluster all over the world [39,80,81].

### 3.5. Putative Genes of Polysaccharide and Biosurfactant Biosynthesis

Bacteria of the genus *Pseudomonas* are known as producers of biosurfactants, which mainly belong to the class of rhamnolipids, glycolipid surface-active molecules that have potential biotechnological applications. The genetic aspects of rhamnolipid production have been most thoroughly studied in the opportunistic pathogen *P. aeruginosa* strain PAO1; its suggested function in this strain is a virulence factor [82]. However, it has been shown that other strains of this species can be successfully used as producers of rhamnolipid biosurfactants, which reduce interfacial tension at the oil–water interface, promoting the detachment of oil from the rock and thereby increasing oil recovery [35].

Most of the species in the genus *Ectopseudomonas* (*P. oleovorans* clade) are not well-known producers of biosurfactants. At the same time, there is information on the production of rhamnolipids by some strains of *E. guguanensis* [83,84,85]. However, for the strains described in these studies, there is no data on the genetic structures that determine the synthesis of the studied biosurfactants. The physiological analysis conducted in this study indicated a significant reduction in the interfacial tension of the liquid culture medium during the growth of strain G3 on crude oil. According to the BlastKOALA portal data, the G3 genome contains annotated genes of oppositely directed operons *rfb*BACD (ACMHYQ_02260-02275) and *rfb*BDAC (ACMHYQ_02230-02215) encoding the biosynthesis of dTDP-L-rhamnose, which is part of rhamnolipids as the primary sugar component. However, since the G3 genome does not contain annotated genes for rhamnolipid biosynthesis (*rhl*ABC), its ability to produce rhamnolipids is questionable. At the same time, the presence of the unique tandem of *gmd*-*tld* genes, which determines the biosynthesis of the rare sugar GDP-6-deoxy-D-talose, was found in the G3 genome (Figure S9). This suggests the possibility that strain G3 may produce a specific biosurfactant in which GDP-6-deoxy-D-talose could theoretically serve as a precursor for sugar components. This assumption requires special research, but its indirect confirmation may be the results of the analysis of the complex of biosurfactants produced by the strain *Rouxiella* sp. DSM 100043, with the most hydrophilic glycolipids containing talose as the carbohydrate moiety [86]. Thus, the study of the biosynthesis of biosurfactants produced by the strain G3 and their genetic determinants requires further special research.

One of the most common exopolysaccharides in bacteria of the genus *Pseudomonas* is alginate, which has been extensively studied in *P. aeruginosa*, where it is a key virulence factor [87]. Alginate is a linear, negatively charged exopolysaccharide composed of β-D-mannuronic acid residues and its C_5_-epimer α-L-guluronic acid (G), linked by 1-4 glycosidic bonds, which plays an important role in biofilm formation [87]. The main genes of alginate biosynthesis in *P. aeruginosa* are organized in the operon *alg*D-*alg*8-*alg*44-*alg*KEGXLIJFA, in which the *alg*D gene encodes GDP-mannose 6-dehydrogenase (EC: 1.1.1.132), a key enzyme in the synthesis of the precursor (GDP-mannuronic acid). The available research literature does not yet contain specific studies focusing on alginate production by the genus *Ectopseudomonas* (*P. oleovorans* clade).

**Figure 5:**
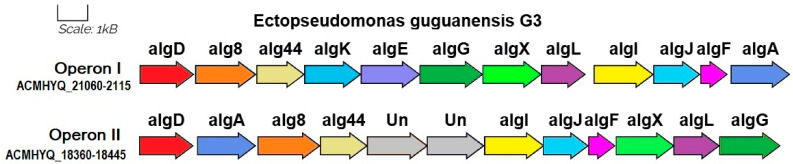
The genes presumably encoding alginate biosynthesis in the G3 genome. Abbreviations: *alg*D, GDP-mannose 6-dehydrogenase; *alg*8, alginate polymerase/glycosyltransferase Alg8; *alg*44, alginate polymerization protein Alg44, membrane fusion protein; *alg*K, alginate export system AlgK/AlgE, periplasmic component AlgK; *alg*E, alginate export system AlgK/AlgE, outer membrane porin AlgE; *alg*G, poly(beta-D-mannuronate) C_5_ epimerase AlgG; *alg*X, alginate O-acetyltransferase AlgX, periplasmic; *alg*L, alginate lyase AlgL; *alg*I, probable poly(beta-D-mannuronate) O-acetylase; *alg*J, alginate O-acetyltransferase AlgJ, inner membrane; *alg*F, alginate O-acetyltransferase AlgF, periplasmic; *alg*A, mannose-1-phosphate guanylyltransferase/mannose-6-phosphate isomerase; Un, unknown. Homologous genes are highlighted in one color.

According to the BlastKOALA portal data, strain G3 is presumably capable of producing all alginate biosynthesis enzymes (Figure S9). However, unlike *P. aeruginosa*, the G3 genome contains annotated genes of two *alg*-operons located in different regions of the genome (Figure 5).

Operon I, *alg*D-*alg*8-*alg*44-*alg*KEGXLIJFA (ACMHYQ_21050-21110), was homologous to the *P. aeruginosa* operon, and the *alg*U gene (ACMHYQ_14090) was that of the AlgU sigma factor for gene expression, and the *muc*ABC genes (ACMHYQ_14095-14105) are negatively regulated by the anti-sigma factor that prevents alginate overproduction. Operon II had a different structure, *alg*DA-*alg*8-*alg*44-Un-Un-*alg*IJFXLG (ACMHYQ_18360-ACMHYQ_18415), and the functions of some genes in it were not identified, so its functionality is less likely than that of operon 1. A double set of potential alginate biosynthesis operons, apart from strain G3, were identified only in the genomes of *E. guguanensis* strains Botswana-61 and HMFL31.

The potential ability of strain G3 to simultaneously produce both alginate and biosurfactants may be important for its survival in oil habitats. Some studies suggest that bacterial strains capable of producing both extracellular polymers (EPS), such as alginate, and biosurfactants simultaneously often exhibit significantly higher efficiency in biofilm formation, resistance to external factors (temperature and salinity), and bioremediation, compared to strains producing only one of these components [88,89].

### 3.6. Genes of Nitrogen Metabolism

Similar to many members of the family *Pseudomonadaceae*, strain G3 is capable of using nitrate (NO_3_^−^) as a nitrogen source for the synthesis of amino acids and other nitrogen-containing compounds (assimilatory nitrate reduction) (Figure S10). The uptake of extracellular nitrate/nitrite into the cell is potentially facilitated by a gene of the NRT2 family (ACMHYQ_17995) encoding an NNP family nitrate/nitrite transporter and the genes *nrt*ABC (ACMHYQ_18025-18035) encoding the ABC cassette nitrate/nitrite transport system (EC: 7.3.2.4). The genes presumably responsible for the assimilatory nitrate reduction process in the G3 genome are *nas*C (ACMHYQ_17975) encoding the assimilatory nitrate reductase catalytic subunit (EC: 1.7.99.4) and *nir*BD (ACMHYQ_17985-17980) encoding the assimilatory nitrite reductase [NAD(P)H] large and small subunits (EC: 1.7.1.4). Adjacent to the genes of assimilatory nitrate reduction is the *cob*A gene (ACMHYQ_17970), encoding uroporphyrin-III C-methyltransferase (EC: 2.1.1.107), a key enzyme in siroheme biosynthesis, which serves as an essential catalytic cofactor for the functioning of assimilatory nitrite reductase [90]. Additional sources of reduced nitrogen compounds in the metabolism of strain G3 may include nitroalkane, the reduction of which to nitrite is presumably catalyzed by the enzyme nitronate monooxygenase (EC: 1.13.12.16), encoded by the *ncd*2 genes (ACMHYQ_22085; 20700), as well as formamide, which is hydrolyzed to ammonia and formate by formamidase (EC: 3.5.1.49), encoded by the *fmd*A gene (ACMHYQ_02750).

Another potential substrate for obtaining reduced nitrogen compounds is urea. Strain G3 is unable to synthesize intracellular urea via the urea cycle due to the absence in its genome of the *arg* gene encoding arginase (EC: 3.5.3.1), which breaks down arginine to ornithine and urea. However, the G3 genome contains annotated genes for several transport systems that facilitate the uptake of urea from the external environment, and these are associated with genes for urea catabolism (Figure S11). The most widespread enzyme in this process is urease (EC: 3.5.1.5), which catalyzes the hydrolysis of urea to ammonia and carbonic acid.

**Figure 6:**
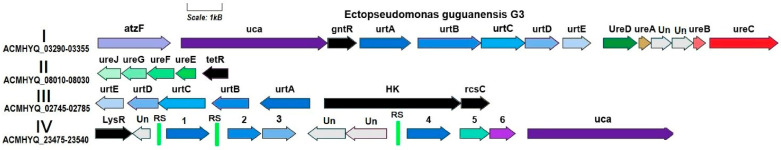
The genes presumably encoding urea degradation and transport in strain G3 genome. Abbreviations: *atz*F, allophanate hydrolase; *uca*, urea carboxylase; *gnt*R, GntR family transcriptional regulator; *urt*A, urea ABC transporter, substrate-binding protein UrtA; *urt*B, urea ABC transporter, permease protein UrtB; *urt*C, urea ABC transporter, permease protein UrtC; *urt*D, urea ABC transporter, ATPase protein UrtD; *urt*E, urea ABC transporter, ATPase protein UrtE; *ure*D, urease accessory protein UreD; *ure*A, urease gamma subunit; *ure*B, urease beta subunit; *ure*C, urease alpha subunit; *tet*R, TetR family transcriptional regulator; *ure*E, urease accessory protein UreE; *ure*F, urease accessory protein UreF; *ure*G, urease accessory protein UreG; *ure*J, nickel-binding accessory protein UreJ-Hup; HK, two-component system sensor histidine kinase/response regulator hybrid; *rcs*C, LuxR family two-component transcriptional response regulator; *lys*R, LysR family transcriptional regulator; 1, urea carboxylase-related ABC transporter, substrate-binding protein; 2, urea carboxylase-related ABC transporter, permease protein; 3, urea carboxylase-related ABC transporter, ATP-binding protein; 4, urea carboxylase-related ABC transporter, substrate-binding protein; 5–6, urea carboxylase-related aminomethyltransferase; RS, guanidine-I riboswitch; Un, unknown. Homologous genes are highlighted in one color.

In the G3 genome, the main urease operon (I) consists of the structural *ure*ABC genes (ACMHYQ_03335-03355), encoding three subunits of urease, and the accessory *ure*D gene (ACMHYQ_03330), encoding the protein that stabilizes the inactive form of the enzyme (Figure 6). The remaining accessory urease genes *ure*EFGJ/*hup*E (ACMHYQ_08025-08010), encoding proteins that contribute to the formation of the enzyme’s active site and the transfer of nickel ions into it, constitute a separate operon (II), which also includes the gene *tet*R (ACMHYQ_08030) for a transcriptional regulator of the TetR family. The main urease operon also includes a cluster of genes *urt*ABCDE (ACMHYQ_03305-03325), encoding ABC-type (ATP-binding cassette) transporters that use energy from ATP to transport urea across the cell membrane. This operon is flanked by a gene for a GntR family transcriptional regulator (ACMHYQ_03300). Another *urt*ABCDE operon (III) is located separately in another region of the genome (ACMHYQ_02775-02755) with the genes of a two-component transcriptional response regulator, LuxR family (ACMHYQ_02780-02785). The alternative pathway for urea degradation occurs via biotin-dependent urea carboxylase (EC: 6.3.4.6) and allophanate hydrolase (EC: 3.5.1.54), encoded by the genes *uca* and *atz*F, respectively [91].

In the G3 genome, the tandem of *atz*F-*uca* genes is annotated, adjacent to the main urease operon (ACMHYQ_03290-03295), as well as an additional *uca* gene located in another region of the genome (ACMHYQ_23535), associated with urea carboxylase-related ABC transporter genes (ACMHYQ_23495-23520) and guanidine-I riboswitches. This operon (IV) is putatively regulated by a LysR family transcriptional regulator (ACMHYQ_23485). Studying similar genetic determinants of urea carboxylase in some *Enterobacteriaceae* previously suggested that the product of the additional *uca* gene, regulated by guanidine-I riboswitches, may function as guanidine carboxylase, using extracellular guanidine as the sole nitrogen source [92]. The whole set of genetic structures related to urea catabolism in the G3 genome is unique for the species *E. guguanensis*. It should be noted that for the functioning of urea carboxylase, the availability of biotin is particularly important; the genes for the complete biosynthetic pathway of the latter are annotated in the G3 genome.

Although the consumption of extracellular urea by strain G3 requires specialized studies, the presence of several systems for its transport into the cell, as well as of the genes responsible for performing both processes of its catabolism, may indicate the importance of this process for the growth of the studied strain. These findings reveal the flexibility of the strain’s nitrogen metabolism and elucidate its ability to grow in nitrogen-poor environments, including formation water of oil reservoirs.

### 3.7. Genes of Osmoprotectant Metabolism

**Figure 7:**
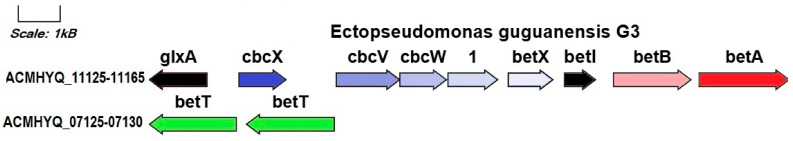
The genes presumably encoding betaine biosynthesis and transport in the G3 genome. Abbreviations: *glx*A, transcriptional regulator; *cbc*X, choline binding ABC transport system substrate-binding protein CbcX; *cbc*V, glycine betaine/L-proline transport ATP-binding protein CbcV; *cbc*W, glycine betaine/L-proline transport system permease protein CbcW; 1, choline-binding protein; *bet*X, betaine ABC transporter, substrate-binding protein BetX; *bet*I, TetR family transcriptional regulator BetI; *bet*B, betaine aldehyde dehydrogenase; *bet*A, choline dehydrogenase; *bet*T, choline transporter.

In addition to the direct transport of betaine from the external environment via the specialized ProP system, the halotolerance of strain G3 is supported by the products of the *bet*IBA gene cluster (ACMHYQ_11155-11165) (Figure 7), encoding choline dehydrogenase (EC: 1.1.99.1) and betaine-aldehyde dehydrogenase (EC: 1.2.1.8), which sequentially oxidize choline to betaine, indicating the potential ability to synthesize betaine. The *bet*I gene is a transcriptional repressor involved in the regulation of betaine biosynthesis by oxygen, choline, and osmotic stress [93]. The betaine synthesis gene cluster is adjacent to the *cbc*XVW genes (ACMHYQ_11130-11140) encoding the choline/glycine betaine ABC transport system for transporting choline from the environment [94] and two additional associated genes: *bet*X (ACMHYQ_11150) encoding a periplasmic solute-binding protein primarily responsible for betaine uptake, and a gene (ACMHYQ_11145) encoding a choline-binding protein. Furthermore, the G3 genome contains two annotated genes, *bet*T (ACMHYQ_07130-07135), encoding a low-affinity but high-capacity choline transporter whose activity is autoinhibited under low-osmolarity conditions and activated by osmotic stress [95,96]. Although some *Pseudomonas* spp. are known for ectoine and hydroxyectoine synthesis due to the *ect*ABCD genes [97,98], the halotolerance of strain G3 is presumably provided solely by betaine production and betaine uptake from the environment.

## 4. Conclusions

Halotolerant hydrocarbon-oxidizing *Ectopseudomonas guguanensis* strain G3 isolated from the oil field with high-salinity formation water was characterized phenotypically and genotypically. The strain is able to grow within a wide range of temperatures and at high NaCl concentrations; growth of strain G3 with crude oil is accompanied by a decrease in surface and interfacial tension of culture liquid, which is of great potential for its application in MEOR technologies. Genomic analysis of the strain confirms its adaptation to the high salinity of the habitat via betaine synthesis and its ability for hydrocarbon degradation including aliphatic (*n*-alkanes) and aromatic (benzoate, catechol, toluene, xylene) compounds, nitrogen consumption via nitrate and urea, presumable alginate and biosurfactant production and, therefore, demonstrates its biotechnological potential. This study is the first investigation of *E. guguanensis* genomics; therefore, it has identified many challenges and opportunities for further research such as gene expression analysis and/or direct quantification of biosurfactant compounds. The application of the *E. guguanensis* strain G3 may be recommended for enhanced oil recovery in reservoirs with high-salinity formation water.

## Figures and Tables

**Table 1:** Rheological characteristics of G3 strain aerobic growth in mineral medium with different carbon sources at 30 °C for 7 days in comparison with sterile medium with the same amount of crude oil used as an abiotic control.

Parameter/Substrate	Control	Glucose	Fumarate	Ethanol	Glycerol	Crude Oil
Surface tension, mN·m^−1^	68.5 ± 0.5	56.2 ± 0.7	57.8 ± 1.7	57.4 ± 0.8	60.7 ± 0.4	46.5 ± 0.7
Interfacial tension, mN·m^−1^	40.0 ± 0.9	16.4 ± 0.3	11.1 ± 1.3	13.0 ± 0.8	9.7 ± 2.4	2.7 ± 0.2

## Data Availability

The 16S rRNA gene sequence and the genomic assembly of G3 strain has been deposited at the GenBank/EMBL/DDBJ under accession numbers PX970282.1 and GCF_047468755.1 (JBLGTS000000000.1), respectively.

## Supplementary Materials

The following supporting information can be downloaded at: https://www.mdpi.com/article/10.3390/biology15120937/s1, Figure S1: Cell morphology of strain G3 grown in TEG medium at 30 °C for 5 days. The samples were examined under a scanning electron microscope (Quattro S, Thermo Fisher Scientific, Brno Černovice, Czech Republic) at an accelerating voltage of 15 kV. Bar, 5 µm; Figure S2: ANI matrix (%) of the *E. guguanensis* strain G3 and phylogenetically closely related whole-genome sequences from GenBank and their cladogram; Figure S3: Distribution of genes in the genome of the *E. guguanensis* strain G3 among subsystem super classes and coverage of the genes in the subsystems; Figure S4: The maximum-likelihood phylogenetic tree derived from 500 single-copy proteins shows the position of strain G3 within the genus *Ectopseudomonas*. Bar: 0.1 amino acid substitutions per site. Bootstrap values > 90 are listed as percentages at the branching points. The tree was rooted using *Pseudomonas aeruginosa* PA14 (GCA_045689255) as the outgroup. Accession numbers for the genomic assemblies are indicated in brackets. The name of the studied strain is marked by red boldface. The cluster of strains of *E. guguanensis* species is marked by a red asterisk; Figure S5: The circular genome map of strain G3 with BLAST comparison with genomes of the of seven other *E. guguanensis* strains. Abbreviations for genes: *rfb*BACD, *rfb*BDAC, clusters of dTDP-L-rhamnose biosynthesis; *glt-tmd*, the tandem of GDP-6-deoxy-D-talose biosynthesis; *fmd*A, formamidase; *ure*ABCD- *urt*ABCDE-1, urease operon I; *uca-atz*F, the tandem urea carboxylase and allophanate hydrolase; *urt*ABCDE, urea transmembrane transporters; *glp*KD_1, glpKD_2, glycerol kinase and glycerol-3-phosphate dehydrogenase; *bet*T1-2, choline transporter; *pro*P, L-proline/glycine betaine transporter; *ure*EFGJ, operon II of accessory urease genes; *ben*IBA-*cbc*XVW, choline dehydrogenase and choline/glycine betaine ABC transporter; *rub*A, *rub*AB, rubredoxin and rubredoxin reductase; *alk*B1, *alk*B2, *alm*A, alkane 1-monooxygenase; NRT2, *nrt*ABC nitrate/nitrite transporters; *nas*C-*nir*BD-*cob*A, the assimilatory nitrate reductase, the assimilatory nitrite reductase, and uroporphyrin-III C-methyltransferase; *alg*-operon_1 and *alg*-operon_2, two putative alginate biosynthesis operons; *ncd*2_1, *ncd*2_2, nitronate monooxygenase; *ben*ABCD/*xyl*XYZL, benzoate/toluate 1,2-dioxygenase and dihydroxycyclohexadiene carboxylate dehydrogenase; *xyl*BAMC, benzyl alcohol dehydrogenase, toluene methyl-monooxygenase, and benzaldehyde dehydrogenase; *xyl*EGFJQKIH, the operon of the catechol *meta*-cleavage pathway; *uce_*2, urea carboxylase; Figure S6: KEGG map of fatty acid degradation pathways based on the genome analysis of strain G3. The enzymes encoded by the genes annotated in the genome are highlighted in green. The presumptive modules for fatty acid beta-oxidation are highlighted in pink and for *n*-alkane oxidation in red; Figure S7: KEGG map of benzoate metabolism pathways based on the genome analysis of strain G3. The enzymes encoded by the genes annotated in the genome are highlighted in green. The presumptive modules of benzoate and catechol degradation are highlighted in red; Figure S8: Localization of benzoate, catechol, and xylene degradation genes in the G3 genome. Genes of mobile elements (ME) are highlighted in green. HGT (horizontal gene transfer) regions are shown according to the results of the Alien Hunter module of the Proksee server (red) and the IslandViewer server (orange). Abbreviations: *ben*A, benzoate 1,2-dioxygenase alpha subunit; *ben*B, benzoate 1,2-dioxygenase beta subunit; *ben*C, benzoate 1,2-dioxygenase, ferredoxin reductase component; *ben*D, 1,2-dihydroxycyclohexa-3,5-diene-1-carboxylate dehydrogenase; *xyl*E, catechol 2,3-dioxygenase; *xyl*G, 2-hydroxymuconate-6-semialdehyde dehydrogenase; *xyl*F, 2-hydroxymuconic semialdehyde hydrolase; *xyl*J, 2-hydroxyhexa-2,4-dienoate hydratase; *xyl*Q, acetaldehyde dehydrogenase; *xyl*K, 4-hydroxy-2-oxovalerate aldolase; *xyl*I, 4-oxalocrotonate decarboxylase; *xyl*H, 2-hydroxymuconate tautomerase; *xyl*S, transcriptional activator protein; xylR, transcriptional regulator protein; *xyl*C, benzaldehyde dehydrogenase (NAD); *xyl*M, toluene methyl-monooxygenase; *xyl*A, toluene methyl-monooxygenase electron transfer component; *xyl*B, aryl-alcohol dehydrogenase; Figure S9: KEGG map of fructose and mannose metabolism pathways based on the genome analysis of strain G3. The enzymes encoded by the genes annotated in the genome are highlighted in green. The presumptive biosynthesis modules are highlighted for GDP-D-mannose in pink, for GDP-6-deoxy-D-talose in red, and for alginate in blue; Figure S10: KEGG map of nitrogen metabolism pathways based on the genome analysis of strain G3. The enzymes encoded by the genes annotated in the genome are highlighted in green. The presumptive modules for assimilative nitrate/nitrite reduction are highlighted in pink and for reduction of other nitrogenous compounds in red; Figure S11: KEGG map of arginine biosynthesis pathways based on the genome analysis of strain G3. The enzymes encoded by the genes annotated in the genome are highlighted in green. The presumptive modules for urea degradation are highlighted in red; Table S1: Comparison of functional genes of alkane oxidation and osmoprotectant metabolism of strain G3 and phylogenetically closely related strains/MAGs of the *E. guguanensis* species.
