# Supplementary material for: Genomic Analysis of the Halotolerant Hydrocarbon-Oxidizing Bacterium Ectopseudomonas guguanensis G3 from a Petroleum Reservoir
Source: Biology (Basel). 2026 Jun 16;15(12):937. doi: 10.3390/biology15120937 (PMC13296017; doi:10.3390/biology15120937)
Supplement: Supplementary file 1 [file biology-15-00937-s001.zip › biology-4316587-supplementary.pdf]

biology

Supplementary Materials

Genomic Analysis of the Halotolerant Hydrocarbon-Oxidizing Bacterium *Ectopseudomonas guguanensis* G3 From a Petroleum Reservoir

Alexey P. Ershov *, Tatyana P. Tourova, Diyana S. Sokolova, Ekaterina M. Semenova and Tamara N. Nazina

Winogradsky Institute of Microbiology, Research Center of Biotechnology, Russian Academy of Sciences, 119071 Moscow, Russia; e.alexey.mail@yandex.ru (A.P.E.); tptour@rambler.ru (T.P.T.); sokolovadiyana@gmail.com (D.S.S.); semenova_inmi@mail.ru (E.M.S.); nazina@inmi.ru (T.N.N.)

* Correspondence: e.alexey.mail@yandex.ru; Tel.: +7-499-135-03-41 (A.P.E.)

This file includes:

Figures S1 to S11

Table S1

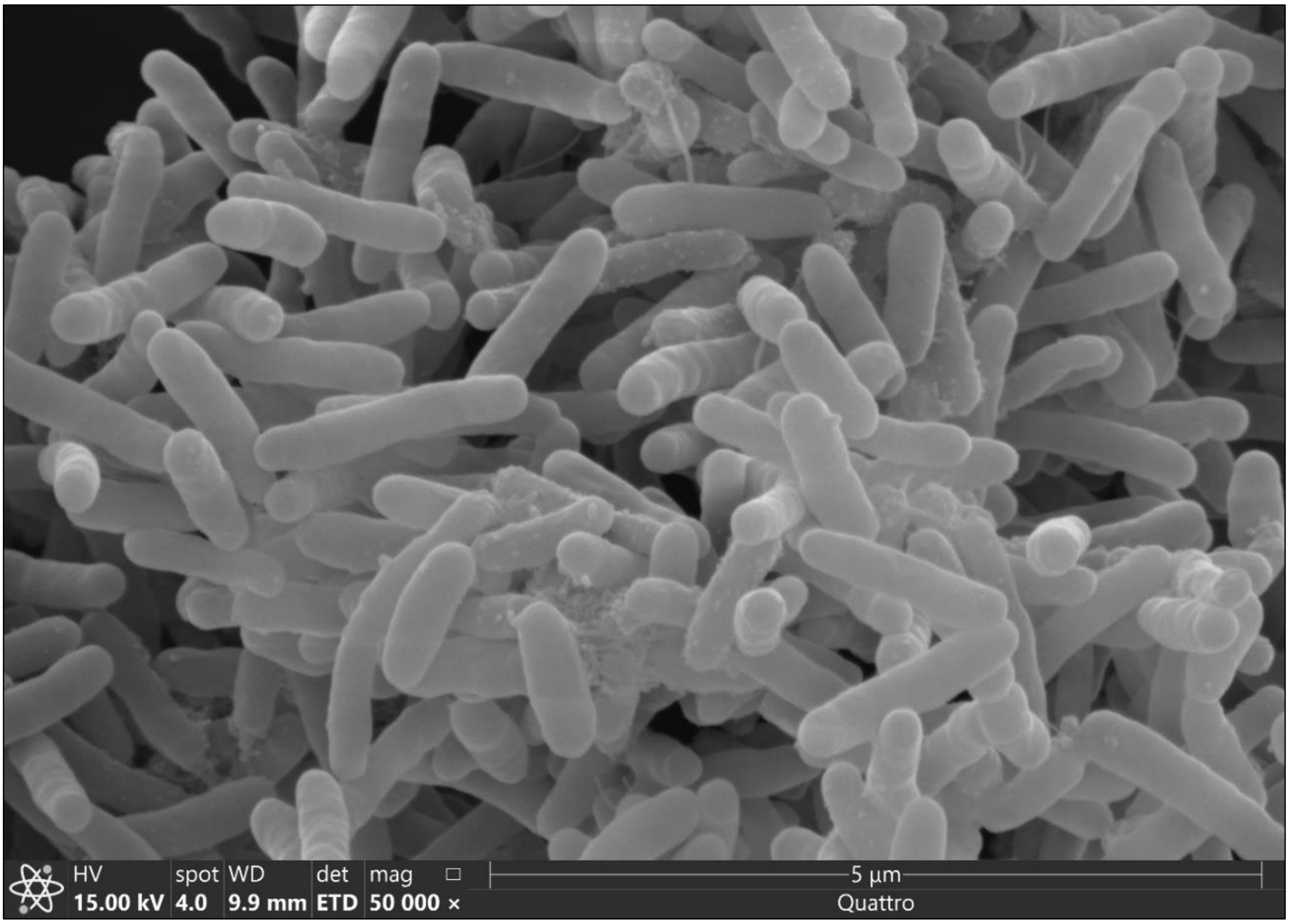

Figure S1. Cell morphology of strain G3 grown in TEG medium at 30 °C for 5 days. The samples were examined under a scanning electron microscope (Quattro S, Thermo Fisher Scientific, Brno Černovice, Czech Republic) at an accelerating voltage of 15 kV. Bar, 5 µm.

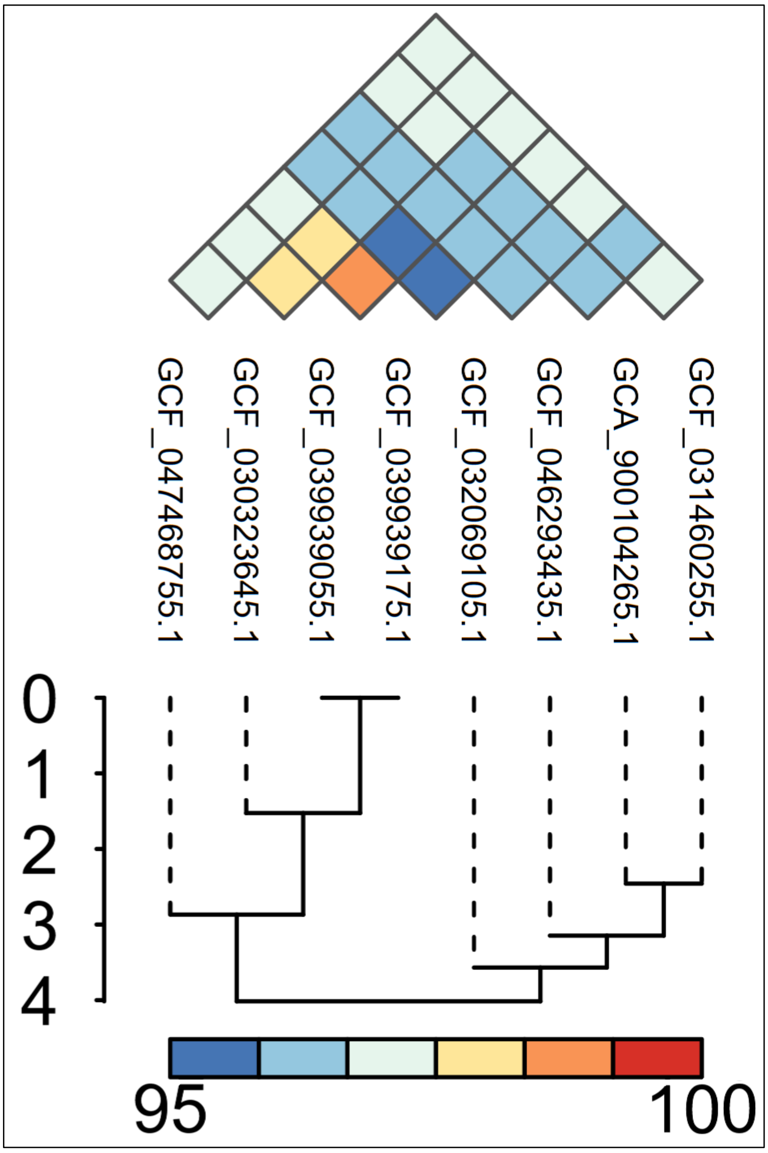

Figure S2. ANI matrix (%) of the *E. guguanensis* strain G3 and phylogenetically closely related whole-genome sequences from GenBank and their cladogram.

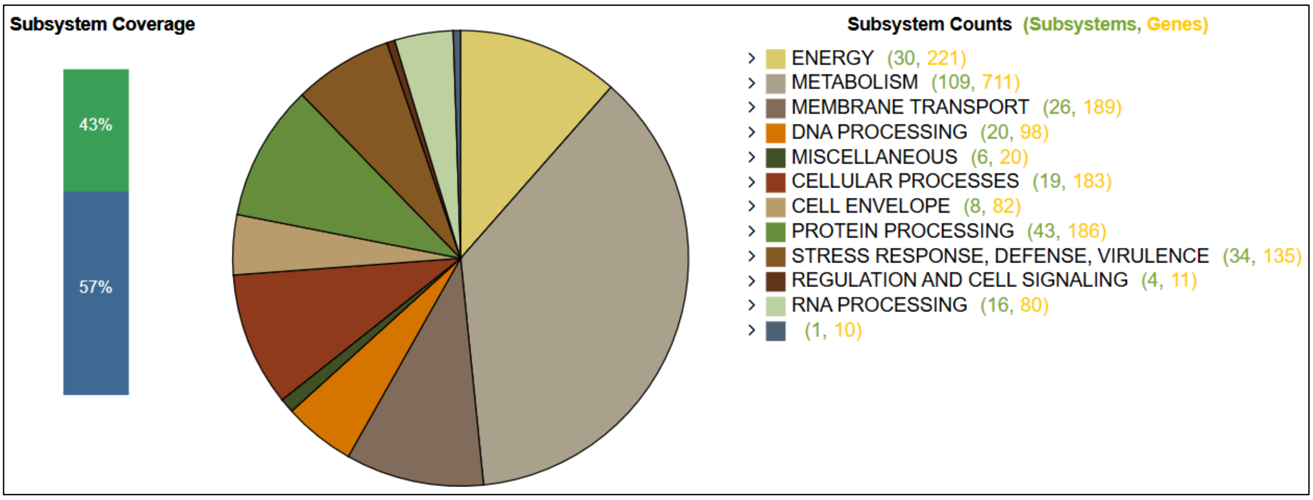

Figure S3. Distribution of genes in the genome of the *E. guguanensis* strain G3 among subsystem super classes and coverage of the genes in the subsystems.

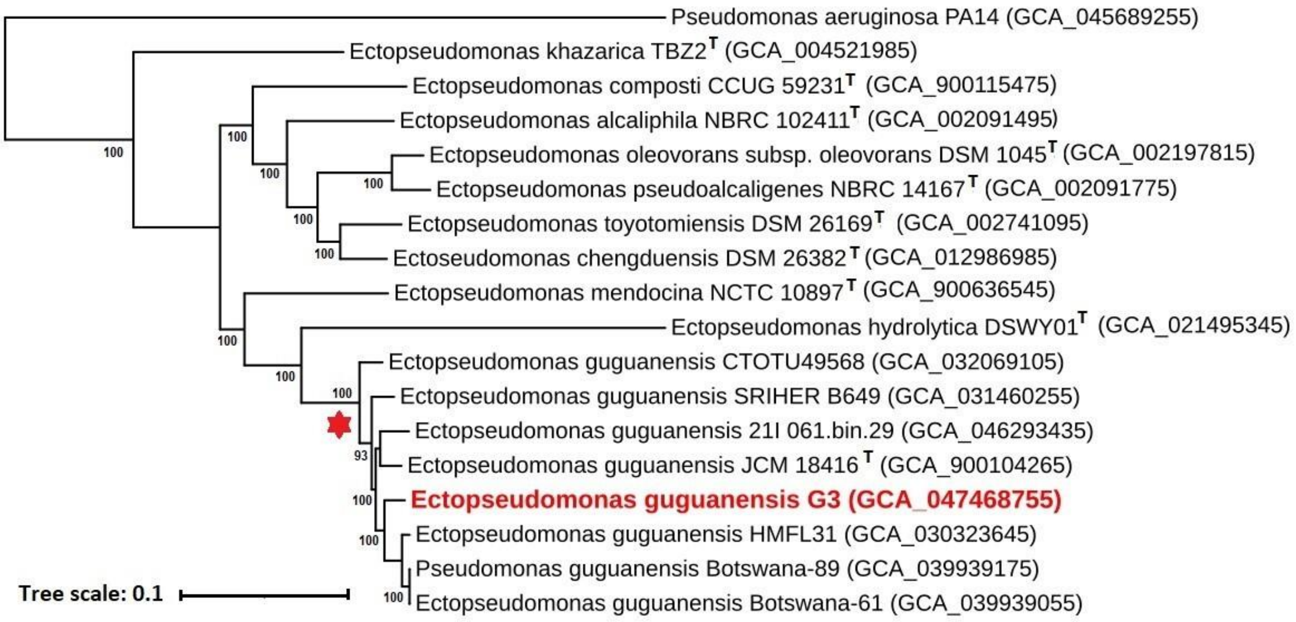

Figure S4. The maximum-likelihood phylogenetic tree derived from 500 single-copy proteins shows the position of strain G3 within the genus *Ectopseudomonas*. Bar: 0.1 amino acid substitutions per site. Bootstrap values >90 are listed as percentages at the branching points. The tree was rooted using *Pseudomonas aeruginosa* PA14 (GCA_045689255) as the outgroup. Accession numbers for the genomic assemblies are indicated in brackets. The name of the studied strain is marked by red boldface. The cluster of strains of *E. guguanensis* species is marked by a red asterisk.

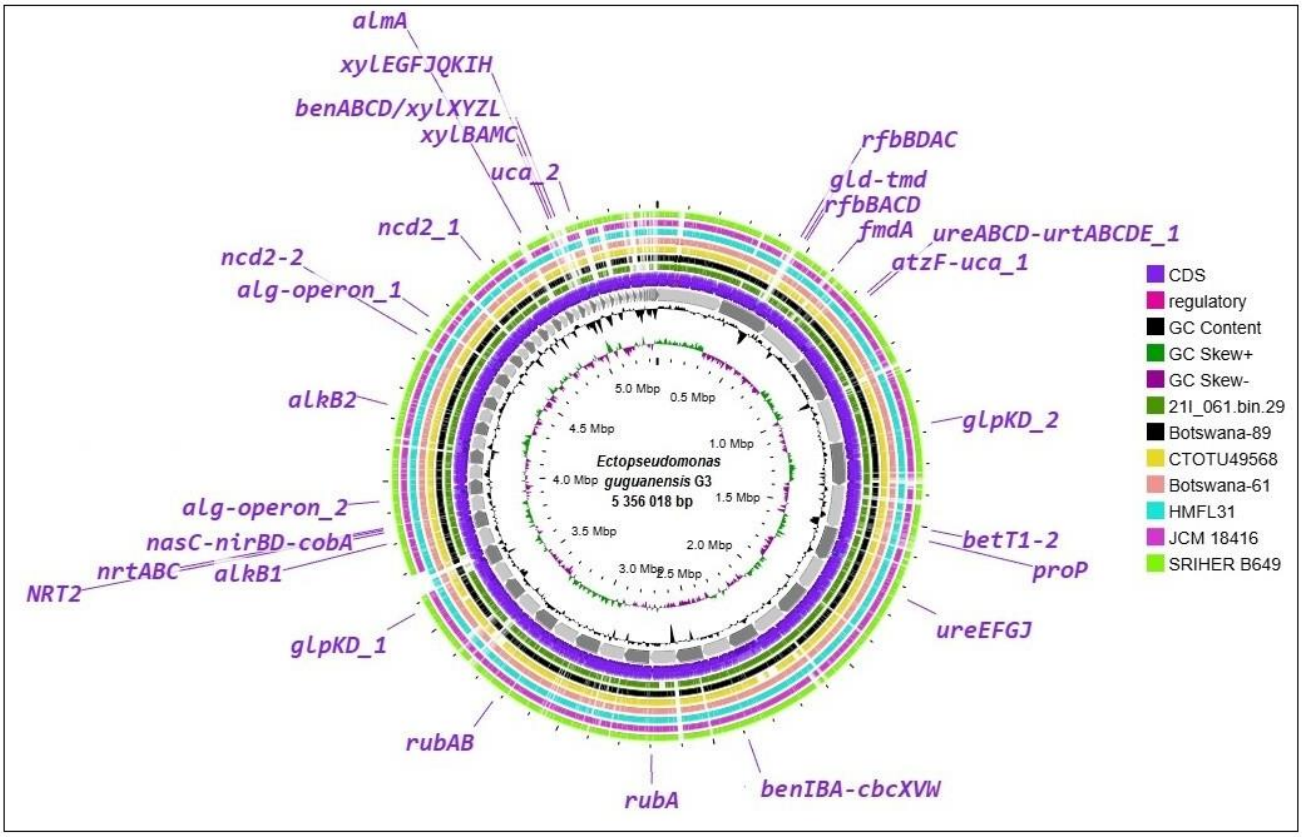

Figure S5. The circular genome map of strain G3 with BLAST comparison with genomes of the of seven other *E. guguanensis* strains. Abbreviations for genes: *rfb*BACD, *rfb*BDAC, clusters of dTDP-L-rhamnose biosynthesis; *glt-tmd*, the tandem of GDP-6-deoxy-D-talose biosynthesis; *fmd*A, formamidase; *ure*ABCD- *urt*ABCDE-1, urease operon I; *uca-atz*F, the tandem urea carboxylase and allophanate hydrolase; *urt*ABCDE, urea transmembrane transporters; *glp*KD_1, glpKD_2, glycerol kinase and glycerol-3-phosphate dehydrogenase; *bet*T1-2, choline transporter; *pro*P, L-proline/glycine betaine transporter; *ure*EFGJ, operon II of accessory urease genes; *ben*IBA-*cbc*XVW, choline dehydrogenase and choline/glycine betaine ABC transporter; *rub*A, *rub*AB, rubredoxin and rubredoxin reductase; *alk*B1, *alk*B2, *alm*A, alkane 1-monooxygenase; NRT2, *nrt*ABC nitrate/nitrite transporters; *nas*C-*nir*BD-*cob*A, the assimilatory nitrate reductase, the assimilatory nitrite reductase, and uroporphyrin-III C-methyltransferase; *alg*-operon_1 and *alg*-operon_2, two putative alginate biosynthesis operons; *ncd*2_1, *ncd*2_2, nitronate monooxygenase; *ben*ABCD/*xyl*XYZL, benzoate/toluate 1,2-dioxygenase and dihydroxycyclohexadiene carboxylate dehydrogenase; *xyl*BAMC, benzyl alcohol dehydrogenase, toluene methyl-monooxygenase, and benzaldehyde dehydrogenase; *xyl*EGFJQKIH, the operon of the catechol *meta*-cleavage pathway; *uce*_2, urea carboxylase.

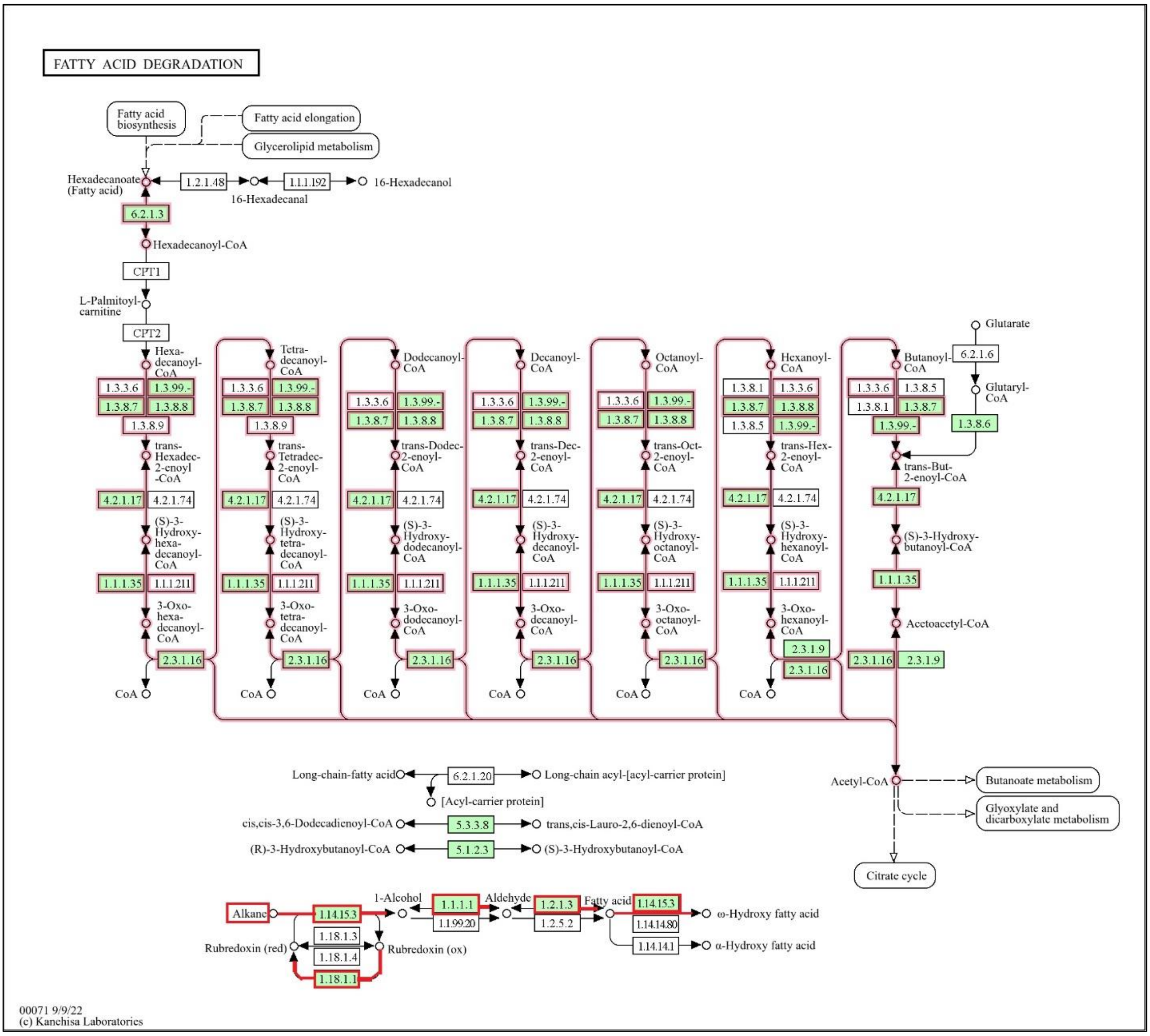

Figure S6. KEGG map of fatty acid degradation pathways based on the genome analysis of strain G3. The enzymes encoded by the genes annotated in the genome are highlighted in green. The presumptive modules for fatty acid beta-oxidation are highlighted in pink and for *n*-alkane oxidation in red.

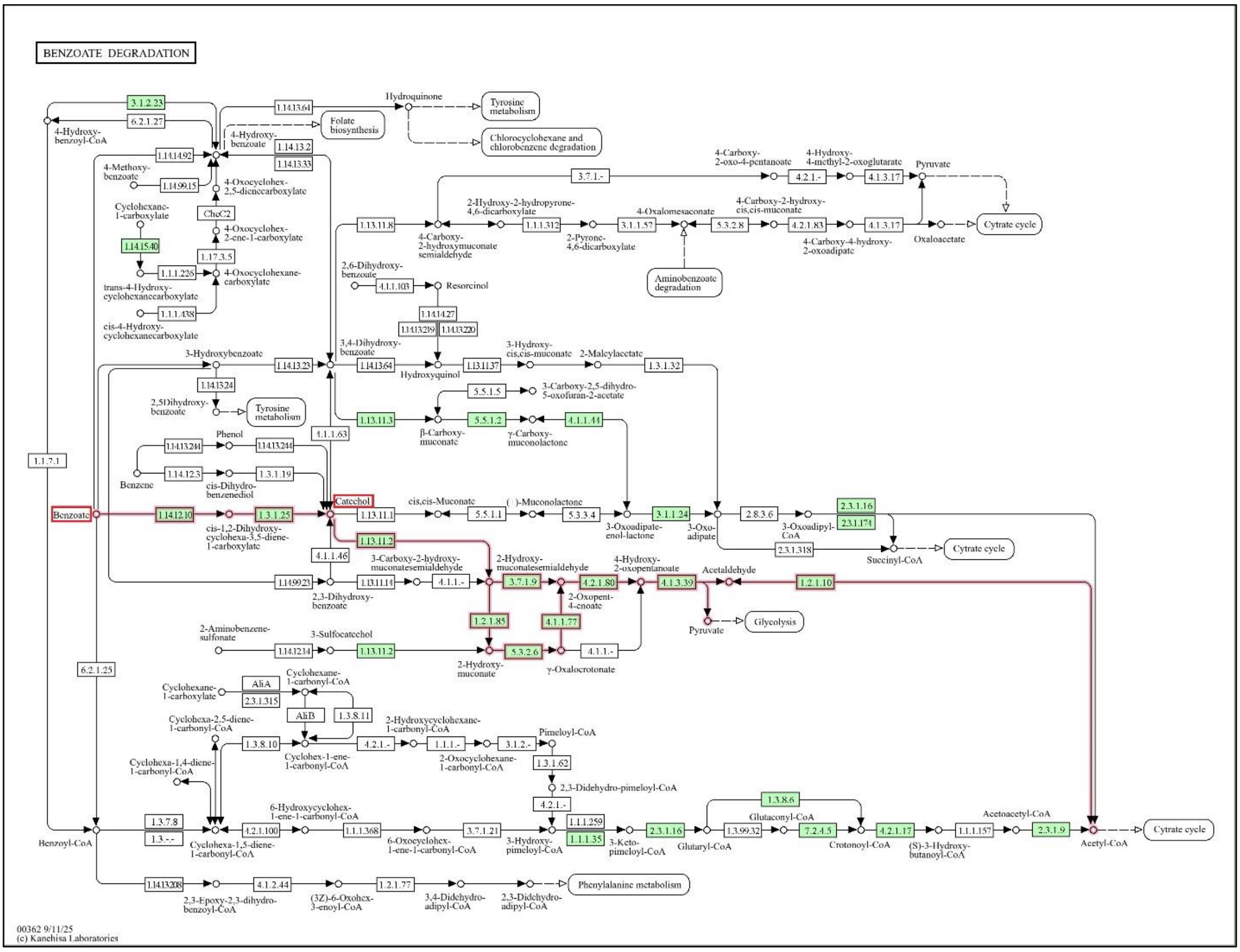

Figure S7. KEGG map of benzoate metabolism pathways based on the genome analysis of strain G3. The enzymes encoded by the genes annotated in the genome are highlighted in green. The presumptive modules of benzoate and catechol degradation are highlighted in red.

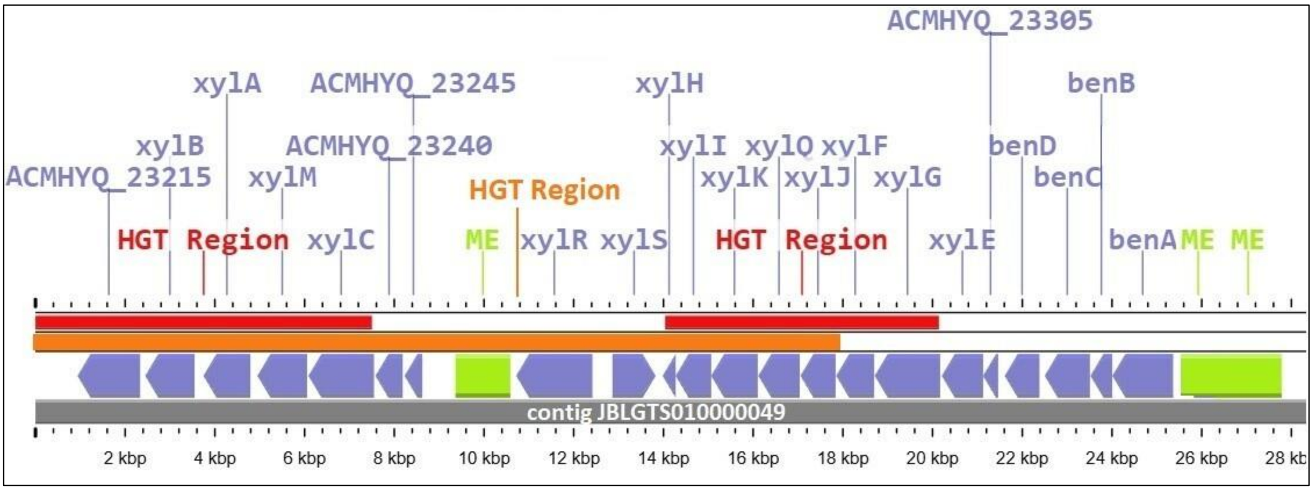

Figure S8. Localization of benzoate, catechol, and xylene degradation genes in the G3 genome. Genes of mobile elements (ME) are highlighted in green. HGT (horizontal gene transfer) regions are shown according to the results of the Alien Hunter module of the Proksee server (red) and the IslandViewer server (orange). Abbreviations: *ben*A, benzoate 1,2-dioxygenase alpha subunit; *ben*B, benzoate 1,2-dioxygenase beta subunit; *ben*C, benzoate 1,2-dioxygenase, ferredoxin reductase component; *ben*D, 1,2-dihydroxycyclohexa-3,5-diene-1-carboxylate dehydrogenase; *xyl*E, catechol 2,3-dioxygenase; *xyl*G, 2-hydroxymuconate-6-semialdehyde dehydrogenase; *xyl*F, 2-hydroxymuconic semialdehyde hydrolase; *xyl*J, 2-hydroxyhexa-2,4-dienoate hydratase; *xyl*Q, acetaldehyde dehydrogenase; *xyl*K, 4-hydroxy-2-oxovalerate aldolase; *xyl*I, 4-oxalocrotonate decarboxylase; *xyl*H, 2-hydroxymuconate tautomerase; *xyl*S, transcriptional activator protein; xylR, transcriptional regulator protein; *xyl*C, benzaldehyde dehydrogenase (NAD); *xyl*M, toluene methyl-monooxygenase; *xyl*A, toluene methyl-monooxygenase electron transfer component; *xyl*B, aryl-alcohol dehydrogenase.

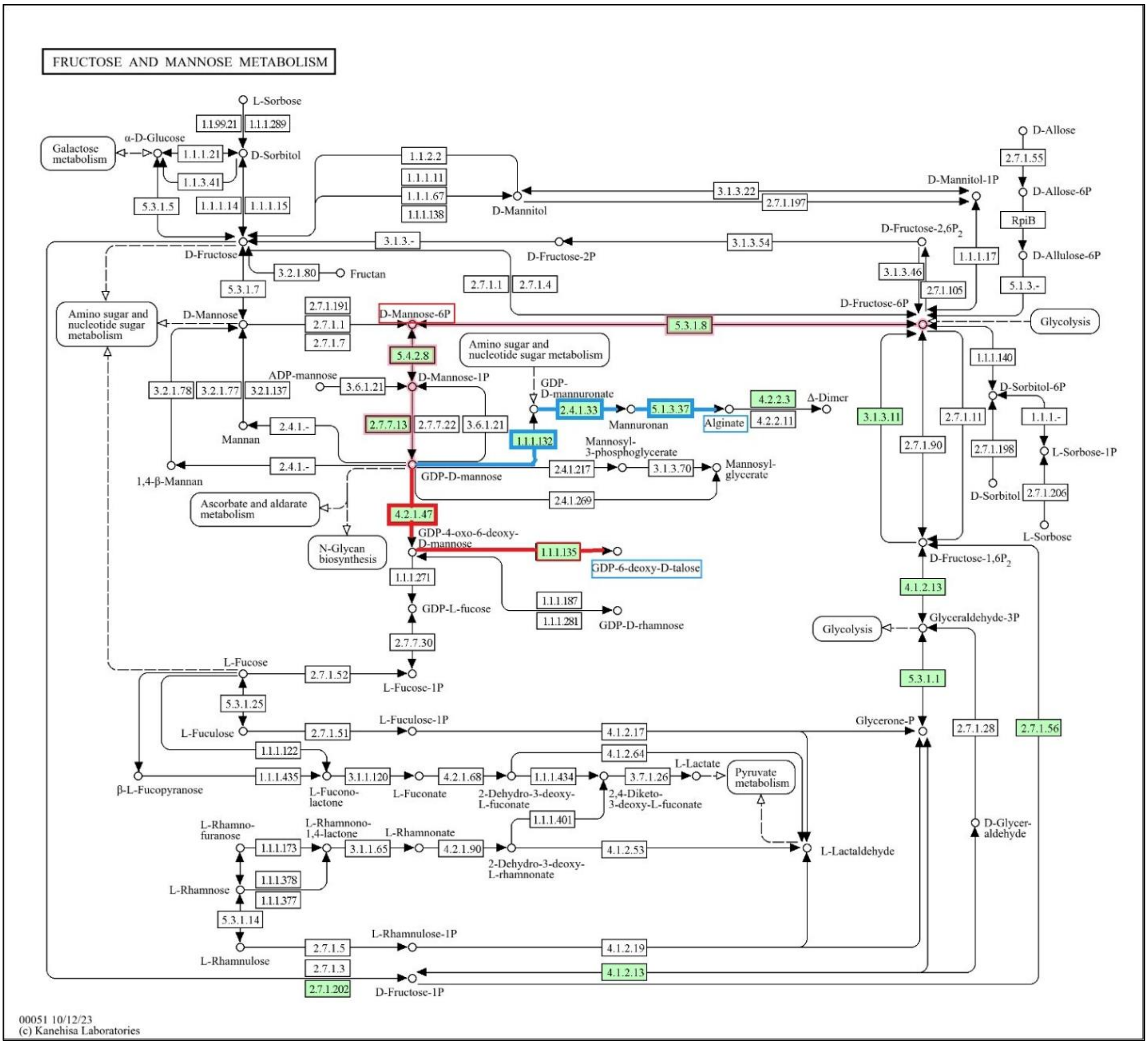

Figure S9. KEGG map of fructose and mannose metabolism pathways based on the genome analysis of strain G3. The enzymes encoded by the genes annotated in the genome are highlighted in green. The presumptive biosynthesis modules are highlighted for GDP-D-mannose in pink, for GDP-6-deoxy-D-talose in red, and for alginate in blue.

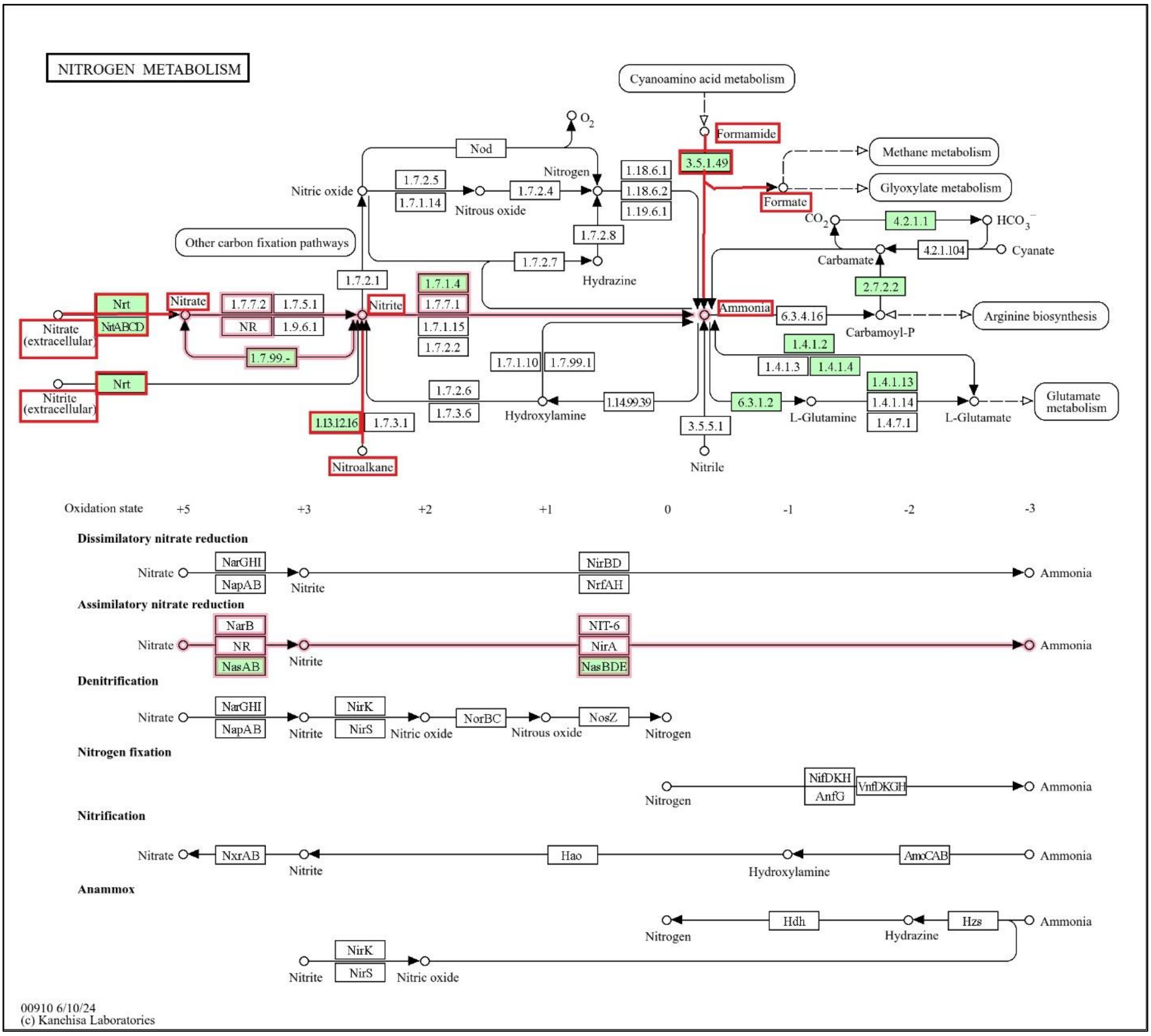

Figure S10. KEGG map of nitrogen metabolism pathways based on the genome analysis of strain G3. The enzymes encoded by the genes annotated in the genome are highlighted in green. The presumptive modules for assimilative nitrate/nitrite reduction are highlighted in pink and for reduction of other nitrogenous compounds in red.

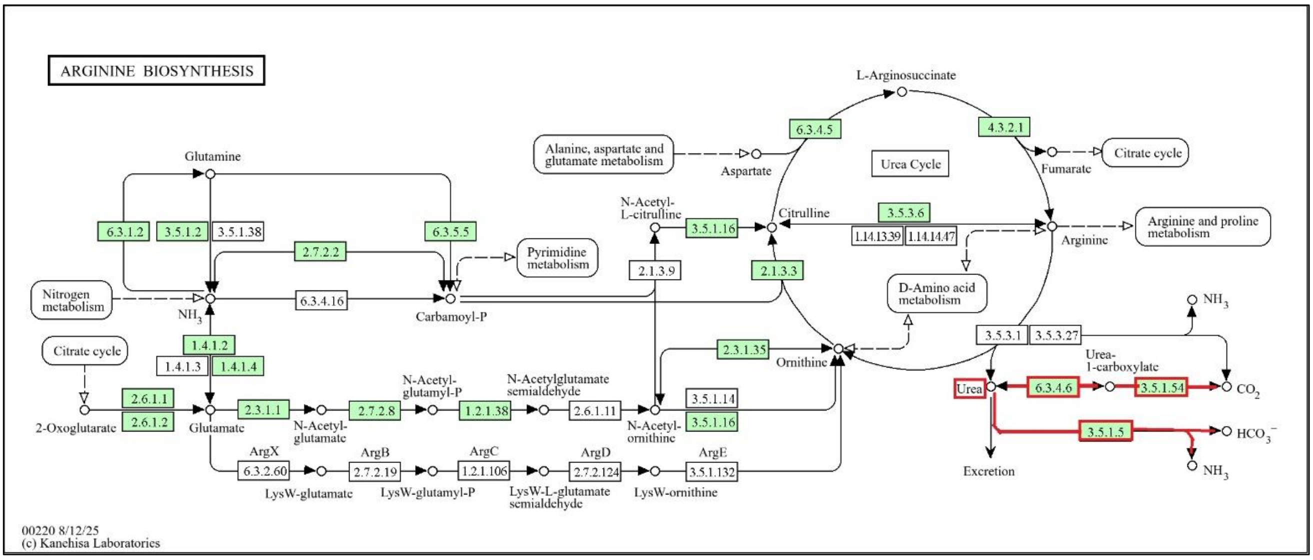

Figure S11. KEGG map of arginine biosynthesis pathways based on the genome analysis of strain G3. The enzymes encoded by the genes annotated in the genome are highlighted in green. The presumptive modules for urea degradation are highlighted in red.

Table S1. Comparison of functional genes of alkane oxidation and osmoprotectant metabolism of strain G3 and phylogenetically closely related strains/MAGs of the *E. guguanensis* species.

**Strain/MAG***	**Genome assembly**	**Status**	**Isolation source**	**Geographic location**	**Annotated functional genes**
21I_061.bin.29*	GCF_046293435.1	WGS	Hot spring water	China: Tibet	*alk*B2, *ben*ABCD, *bet*AB
Botswana-61	GCF_039939055.1	WGS	Portable suction machine	Botswana: Gaborone	*alk*B1, *alk*B2, *bet*AB
Botswana-89	GCF_039939175.1	WGS	Sink drain	Botswana: Gaborone	*alk*B1, *alk*B2, *bet*AB
CC-G9A^T	GCA_900104265.1	WGS	Hot spring water	Thailand	*alk*B2, *bet*AB
CTOTU49568*	GCF_032069105.1	WGS	Urban	USA	*alk*B2, *ben*ABCD, *bet*AB
**G3**	**GCF_047468755.1**	**WGS**	**Oil reservoir**	**Kazakhstan**	***alk*B1, *alk*B2, *ben*ABCD, *bet*AB, *xyl*CMAB, *xyl*EGFJQKIH**
HMFL31	GCF_030323645.1	Complete	Sludge from paper mill effluent treatment	Chile: Biobio Region, Arauco	*alk*B1, *alk*B2, *bet*AB, *xyl*EGFJQKIH
SRIHER B649	GCF_031460255.1	WGS	Oil-contaminated marine water	India: Chennai Harbour	*alk*B1, *alk*B2, *ben*ABCD, *bet*AB
